# Patients’ experiences of a standardized care pathway for suspected bladder cancer due to macroscopic hematuria

**DOI:** 10.1186/s12894-025-01898-1

**Published:** 2025-08-23

**Authors:** Lisa Karlsson, Anette Ek-Steinum, Viola Nyman, Suleiman Abuhasanein

**Affiliations:** 1 Department of Surgery, Urology section, NU Hospital Group, Region Västra Götaland, Uddevalla, Sweden; 2 Department of Health Sciences, University West, Trollhättan, Sweden; 3 Institute of Health and Care Sciences, Sahlgrenska Academy, Gothenburg University, Göteborg, Sweden; 4 Department of Research and Development, NU-Hospital Group, Trollhättan, Sweden; 5 Department of Urology, Institute of Clinical Science, Sahlgrenska Academy, University of Gothenburg, 413 90 Göteborg, Sweden

**Keywords:** Hematuria, Urinary bladder cancer, Interview, Content analysis, Patients' experiences

## Abstract

**Objective:**

To explore patients’ experiences navigating the Standardized Care Pathway (SCP) for macroscopic hematuria through semi-structured interviews.

**Methods and materials:**

An interview study was conducted to explore patients’ experiences of SCP for macroscopic hematuria. The study employed content analysis with an inductive approach, as described by Lundman and Graneheim, to explore both explicit and implicit patterns in the data. Informants were recruited from an outpatient clinic for investigation of macroscopic hematuria, with interview questions derived from literature and clinical experience. Researchers immersed themselves in the data through repeated readings of transcripts, identifying meaning units that were coded and analyzed to develop subcategories reflecting similarities and differences.

**Results:**

Twelve patients, with a median age of 71 years (58% women), participated in the study. Among them, one was diagnosed with urinary bladder cancer (UBC). The findings highlight a mix of appreciation and questioning associated with the SCP process, as patients valued the efficiency of one stop policy for testing but also experienced heightened anxiety. A lack of detailed and patient-centred communication emerged as a key issue, with primary care centers providing insufficient information.

**Conclusions:**

Although SCP is effective, it prompts consideration of whether it is suitable for all patients. A more individually tailored approach might be more appropriate, prioritizing rapid evaluation for those with a high likelihood of cancer while directing others to a standard diagnostic route. Continuing the one-stop policy was seen as beneficial.

**Supplementary Information:**

The online version contains supplementary material available at 10.1186/s12894-025-01898-1.

## Introduction

Urinary bladder cancer (UBC) is among the most common urological cancers worldwide, with an estimated 573 000 new cases and 213 000 deaths in 2020 [1]. The largest risk factors for UBC are smoking, being male, and older age [2, 3]. Blood in urine (known as macroscopic haematuria) is most common alarm symptom of UBC [4]. Generally, in cancer, a delay in diagnosis has a negative impact on prognosis [5]. Therefore, in Sweden, a standardized care pathway (SCP) as a fast track for patients with suspected UBC, mainly due to macroscopic hematuria, was implemented in 2016, with the ambition to improve management and outcome [6].

Patients undergoing cancer diagnostic evaluations frequently face considerable anxiety and a reduced quality of life during the period of uncertainty before receiving a definitive diagnosis [7]. Management of macroscopic hematuria - bearing in mind that one may have UBC- is naturally a stressful process [8]. In Sweden, it is well-documented that patients often face prolonged waiting times before accessing specialist care [9]. The SCP was introduced in part to address these delays by expediting the diagnostic process. However, it remains unclear whether the rapid pace of the SCP alleviates or potentially introduces new stressors for patients. This makes it particularly important to investigate how patients experience the SCP and its impact on their well-being.

While the quality of life (QoL) of UBC patients has been well-described [10], a description of patients’ experiences during the diagnosis process is missing. Understanding this phase is important, as previous studies show that integrating QoL assessments in clinical settings can improve communication, symptom awareness, and even survival outcomes [11]. To our knowledge, no research has been conducted which has exclusively involved interviews with individuals undergoing a process of SCP for macroscopic hematuria. To fill this knowledge gap, the aim of this study was to explore how patients with macroscopic hematuria experience the SCP, with particular attention to their emotional responses, perceived clarity of information during the diagnostic process, and the overall impact of that process. This was done through qualitative analysis of semi-structured interviews.

## Materials and methods

An interview study was conducted to explore patients’ experiences of SCP for macroscopic hematuria. This study was conducted at a large regional hospital (catchment area 320 000 inhabitants) that serves a diverse patient population representative of the general demographic and clinical characteristics seen in Sweden, making the findings relevant to similar healthcare settings internationally. Furthermore, describing how experiences are articulated within this cohort of patients may provide valuable insights for healthcare professionals worldwide working in the field of urology. Throughout this study, the term “informants” is used to refer to the patients. We reviewed the COREQ (Consolidated Criteria for Reporting Qualitative Research) guidelines to ensure thorough and transparent reporting of data collection and analysis processes (Supplementary.1) [12].

### Standardized care pathway

In Sweden, the SCP for macroscopic hematuria was developed using a standardized protocol that involved cystoscopy and computed tomography urography (CTU) for all eligible patients aged 50 and older [13]. The goal is to complete these investigations in a single day, with the CTU performed before the cystoscopy. While this approach is not consistently implemented across the country, our clinic is one of the locations that follows this policy, offering a one-stop policy where both CTU and cystoscopy are completed on the same day in most cases. The target maximum lead time for the investigation of macroscopic hematuria is set at 7 days from referral to the first urology visit, and 13 days from referral to diagnosis, defined as the date of transurethral resection of bladder tumour (TURBT). The aim of the SCP was to enhance patient outcomes, primarily by reducing the time from the onset of symptoms to the initiation of treatment.

### Informants and data collection

A total of 12 Informants were interviewed. The sample size was not predetermined but guided by the principle of data saturation [14]. Interviews were conducted until no new themes or significant insights emerged, indicating that saturation had been reached. This approach aligns with established practices in qualitative research.

Individual interviews were conducted beginning with an open-ended question: *“Can you start by describing the first time you noticed blood in your urine?”* (the interview guide - Supplementary.2) The data was analyzed without preconceived conditions, allowing the informants’ experiences to take centre stage. The semi-structured interviews were conducted in person by one of the authors of this study (AE) and lasted approximately 40 min per interview. All interviews were recorded and transcribed verbatim for analysis by a research assistant (AD).

To provide context for the results, general medical and demographic questions were asked at the start of each interview. Informants were recruited through the urology outpatient clinic at a hospital in Western Sweden. The interview guide was broadly designed by the research team to query informants’ experience and perceptions of medical and social aspects of possibility of suffering of cancer. Interviews included open-ended questions to encourage the dialogue with follow-up questions. Interview questions were developed from the existing literature and authors clinical experience. All patients were initially informed of the intent of the study and the anonymous processing. All patient signed an informed consent to participate in the study.

### Analysis

The data was analyzed using content analysis with an inductive approach, as outlined by Lundman & Graneheim [15]. This method is particularly relevant for qualitative studies, as it allows for the examination of both the manifest and latent content, emphasizing variations in the text and identifying patterns [15]. According to Gillham [16], interviews are a well-established method for obtaining direct insight into informants’ experiences, feelings, and thoughts about the studied phenomenon—*the rapid diagnostic process for potential cancer following the appearance of blood in the urine*.

The transcribed interview material was read multiple times to deepen the understanding of its content and to further immersion in, and familiarity with, the data. The analysis was initiated by identifying meaning units within the text. These units were then coded based on their content. The codes were analysed to create subcategories, highlighting similarities and differences. To illustrate the findings in the study, relevant quotes from the interviews were included in the results.

## Results

### Informants

**Fig. 1:**
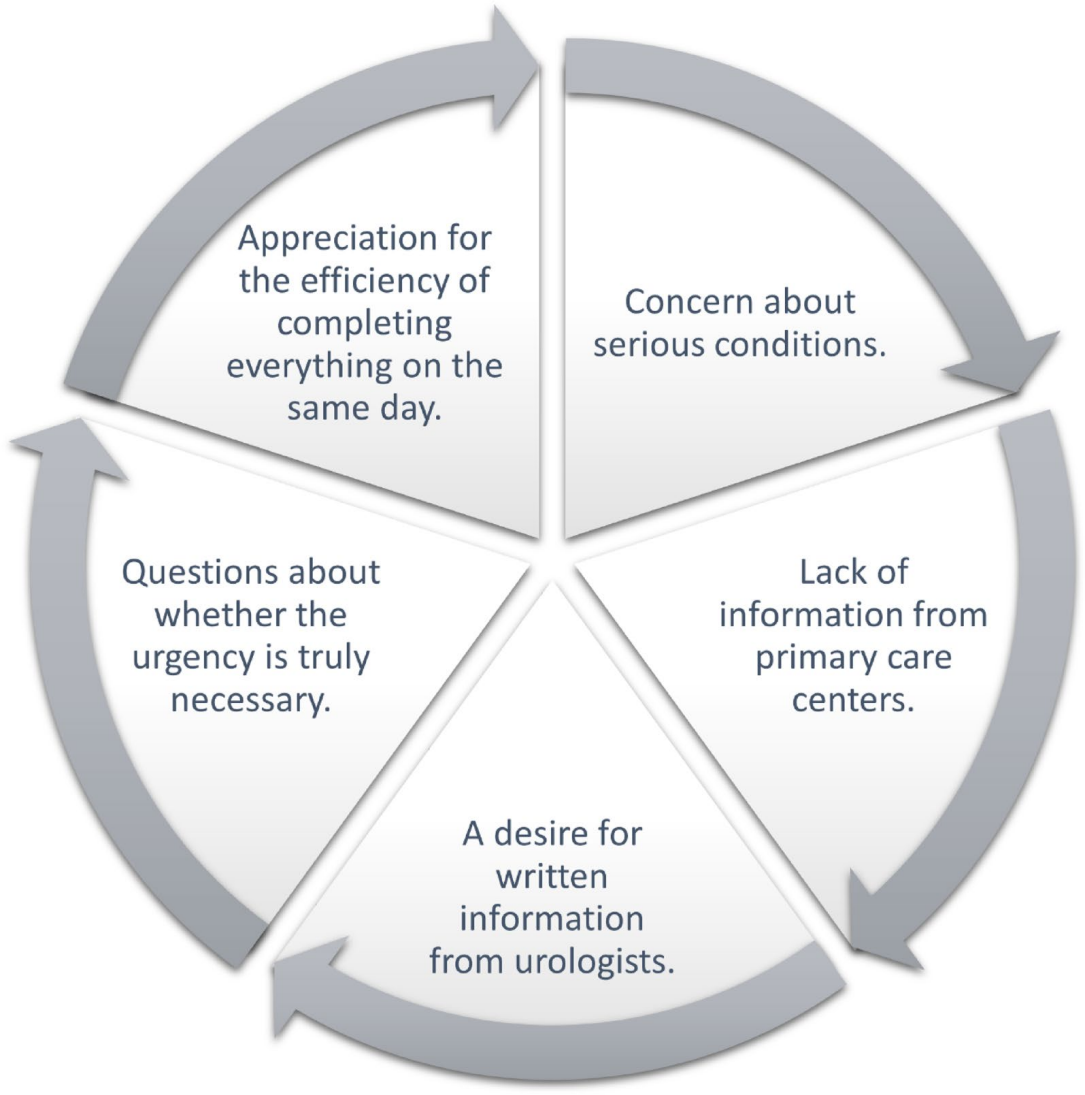
The five identified key categories

This study explored patients’ experiences of the standardized care pathway for macroscopic hematuria. Thematic analysis revealed five key themes reflecting the emotional and informational journey: *Anxiety and uncertainty*, *Insufficient information*, *Quick contact with the urological unit*, *One-stop policy*, and *Is it really urgent?*, highlighting both reassurance and distress (Fig. 1)

Eighteen eligible informants were invited to participate in the study; six declined, while twelve agreed to take part. The final sample consisted of twelve informants, of whom 7 (58%) were women, with a median age of 71 years (interquartile range 65–76). One patient was diagnosed with UBC following macroscopic hematuria, while the others had benign causes for their macroscopic hematuria (Table 1). Patients were selected from those who visited the outpatient clinic for investigation of macroscopic hematuria between April and November 2023and the interviews were conducted three weeks (IQR 1–7) following the initial investigation.

**Table 1:**
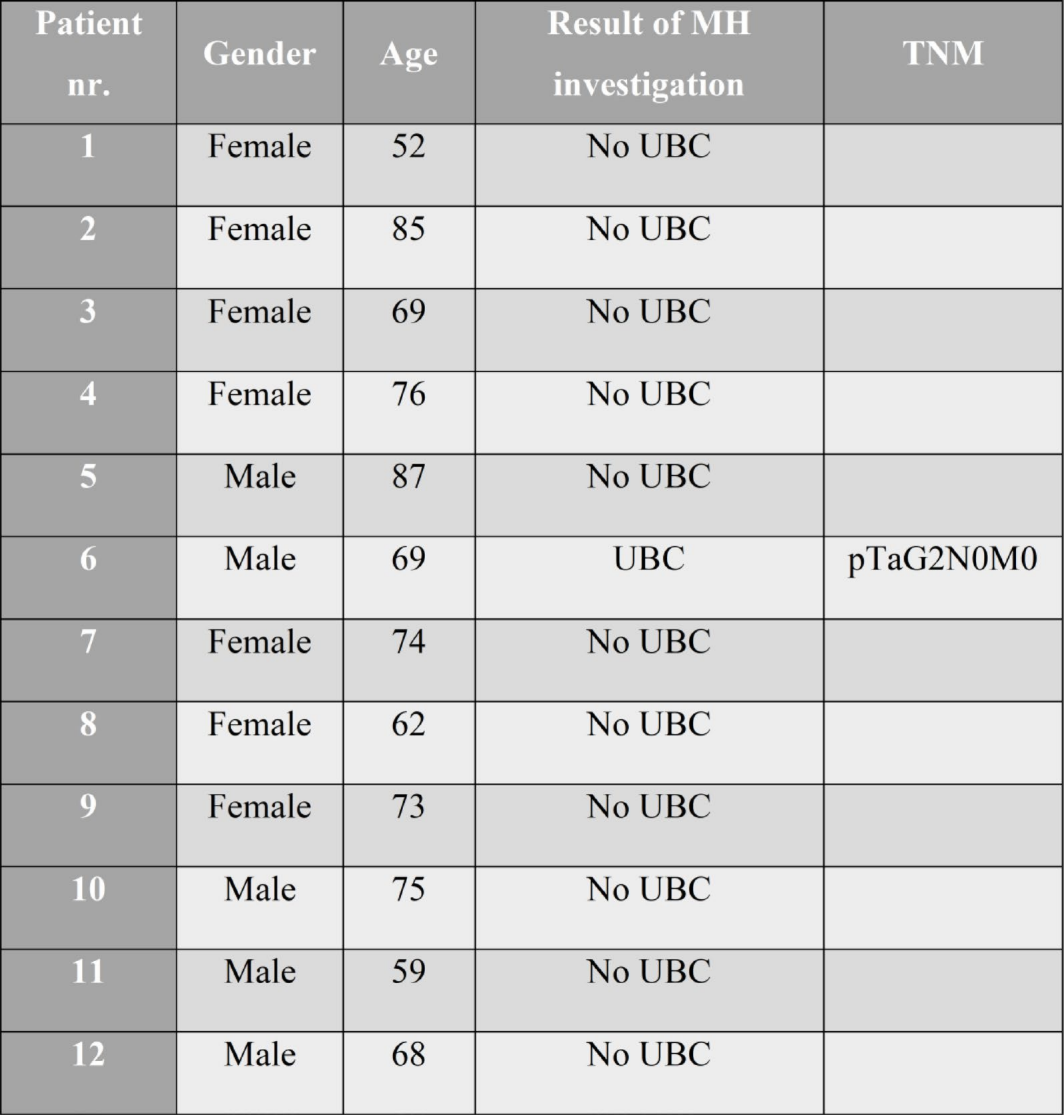
Demographics and clinical features of the informants (MH: macroscopic hematuria - TNM: tumor, node, metastasis - UBC: urinary bladder cancer)

### Anxiety and uncertainty

Informants found seeing blood in their urine for the first time alarming, often leading to fear and visits to the emergency department, especially if the bleeding persisted or worsened. This period was marked by anxiety and uncertainty, which was intensified with additional symptoms such as painful urination, heavier bleeding, or fever.

“I went to the bathroom and…saw some blood, and then…of course, I got scared and went to the emergency department.” Informant-11.

“I thought maybe I had a kidney stone.” Informant-2.

Some informants associated the blood with a urinary tract infection, particularly if they had experienced it previously. When referred to a urologist, concerns about more serious issues emerged. Patients on blood-thinning medications often attributed the bleeding to their treatment. Phone calls from urological unit to schedule prompt appointments often caused anxiety and raised concerns about potentially serious health issues.

“I always think of the worst; I thought it might be cancer.” Informant-1.

“When I received the call from the urologist,* the thoughts started again—what if…” Informant-1*.

### Insufficient information

Patients’ care typically begins in primary care, where they first present with macroscopic hematuria. Primary care providers conduct the initial evaluation and decide if referral to a urologist is necessary. When referral occurs, it initiates the standardized care pathway (SCP), which sets strict timeframes for further investigations by a urologist, such as imaging and cystoscopy. Understanding this division of roles is important, as many patients’ experiences and expectations are shaped during their initial contact with primary care, which influences how they perceive the subsequent specialist investigations and communications.

Many informants received information about further investigations through primary care, where they were informed about the need for testing.

“I was deeply disappointed with the information provided during the conversation. Do you really think one sits calmly at a kitchen table, taking notes on everything?” Informant-3.

They were informed that further examination was required due to the presence of blood in their urine. Many sought additional clarities by web-searching or discussing symptoms with family members.

“I searched online, and it said it could be anything, like cancer or prostate issues.” Informant-12

However, informants noted variations in the information provided by different primary care centers. Many found it comforting to know that everyone over the age of fifty undergoes similar investigations, but they expressed concern about the lack of explanation for the potential causes of blood in the urine. Details regarding investigations for suspected UBC or prostate cancer were inconsistent, and no one reported receiving an explanation of the SCP process.

“I didn’t know about the cystoscopy. If I had known, I would have been more worried.” Informant-1.

“I didn’t receive an explanation from the primary care centre about why I was being referred to the urologist.” Informant-4.

### Quick contact with the urological unit

Phone calls from the urology unit, intended to align with SCP guidelines for time limits in scheduling appointments, were made without prior notification to patients that they would expect a call during that period defined by the SCP. As a result, unexpected calls made it challenging for informants to fully take in the information provided, particularly if the calls occurred in stressful situations. For example, some were called while in public places like stores, making it difficult to take notes or focus on the information being provided.

“I was scared when I got an urgent call in a situation like this. I had no idea whether the call would come the next day, in four days, or even ten days.” Informant-3.

The rapid pace of events also intensified their anxiety, as the expedited healthcare pathway was often perceived as signalling something urgent or serious. Nonetheless, the absence of follow-up through written communication via some form of digital tool forced them to rely on handwritten notes to capture the details of their appointments.

“There was so much information. I think an email or SMS would be better. You could include a form with details like times, floors, and corridors to make things clearer.” Informant- 3.

Many informants expressed a preference for receiving detailed information via SMS or email, allowing them to review and absorb it at their own pace. The unexpected nature of these calls increased fear and uncertainty, as informants were unaware of when the calls would occur or what they would entail. Due to the volume of information shared, informants found it necessary to take notes during the calls and frequently relied on online resources to understand what to expect. Keeping track of all the details related to hospital visits proved challenging and, at times, overwhelming.

“You feel shaken and scared in such a situation, and then you are suddenly called out of nowhere. I had no idea if it would happen the next day, in four days, five days, or ten days. And there I was, in the middle of life, surrounded by grandchildren when the phone rings.” Informant- 3.

### Is it really urgent?

Concerns about urgency and potential danger were frequently raised. Patients often perceived the rapid referral process as an indication of a potentially severe or life-threatening condition that required immediate medical evaluation.

“When it happened, you got the service of things happening so quickly. Then, of course, it raises the anxiety that it is so urgent. It must be something dangerous….” Informant-7.

This perception occasionally led to unnecessary anxiety because the rapid pace of the process was not always indicative of a serious health risk. Informants often interpreted the expedited timeline as a sign of urgency, which heightened their concerns despite the precautionary nature of the pathway.

“At the primary care centre, she asked me: ‘When you get home, do you have anyone there with you?’ I thought, my goodness, why is she asking me this? It scared me. That is when I really started thinking, ‘Something must be wrong…’” Informant- 1.

Informants compared their experience with other health conditions, which typically involved long waiting times for care. While they valued the rapid pace of the standardized care pathway, many found phone calls to be an ineffective way to convey detailed information during a stressful time. They expressed a preference for clearer, more patient-centered communication. Although cancer was directly mentioned during these calls and the urgency to rule it out was stressed, several informants reported feeling curiosity rather than immediate fear. This response may reflect a natural desire to understand and make sense of uncertainty. Some informants speculated whether the rapid process they experienced was part of a specific project. Primary care staff explained that finding blood in the urine automatically triggered a fast referral to a urologist.

“The doctor said, ‘Everyone over fifty with blood in their urine undergoes this kind of examination,’ and that reassured me a bit…” Informant- 9.

Although the tests were often described as uncomfortable, informants generally found the process to be manageable, particularly those who ultimately did not receive a cancer diagnosis. For these individuals, the relief of a benign outcome overshadowed the temporary discomfort associated with the procedures.

“Once I was told that everything looked fine, all the stress disappeared. Being seen quickly meant I did not have to spend time worrying at home.” Informant- 3.

However, some mentioned that the speed of the process heightened their anxiety rather than relieving it. Informants were given the option to view the screen during the cystoscopy, allowing them to see if they had bladder stones. One informant even learned during the procedure that they had UBC.

### One stop policy

Overall, informants expressed strong appreciation for the promptness of care within the one-stop policy. Although the hospital visit was often accompanied by anxiety and anticipation related to the various diagnostic tests, many felt reassured by the fact that all necessary examinations were completed within a single day. This streamlined approach minimized the need for multiple hospital visits, reducing both logistical burdens and emotional stress. Notably, this rapid and comprehensive evaluation is not yet widely available nationwide, making it especially valued by patients who travelled long distances for their appointments. The ability to receive test results on the same day provided significant relief, shortening the period of uncertainty.

“I found it positive that everything moved so quickly… it all happened in just a few hours. Sure,* it took almost a full day*,* but still… it was amazing how fast things moved from the X-ray to the cystoscopy.” Informant-10*.

This efficient process contributed to a sense of being well cared for despite the challenging circumstances.

## Discussion

The findings highlight the complex nature of patients’ experiences during the diagnostic process for blood in urine, revealing both the advantages and challenges of SCP. Five key categories emerged from their accounts: while the swift diagnostic process provided relief, it also heightened anxiety and stress about potential serious conditions; dissatisfaction with the limited information provided by primary care; a strong desire for written information from urologists; uncertainty about the urgency of the expedited process; and an appreciation for the convenience of completing all tests in a single visit. These findings underscore the pressing need for more comprehensive, patient-centred communication that ensures efficiency is balanced with clear explanations and emotional support.

The duality of gratitude and curiosity about the urgency is a central topic in patients’ experiences during investigations for macroscopic hematuria. Patients often expressed relief and appreciation for the swift diagnostic process, recognizing the value of quickly addressing a potentially significant issue. However, this gratitude was accompanied by a sense of curiosity and questioning about the rationale for such urgency, particularly given the relatively low likelihood of a cancer diagnosis. This paradox highlights the importance of effective communication to explain the rationale behind the expedited pathway, alleviating unnecessary anxiety while maintaining trust in the process. Moreover, tailoring the SCP based on the severity of symptoms or other criteria could further enhance patient satisfaction and efficiency. By distinguishing between cases requiring immediate attention and those that can follow a less urgent pathway, healthcare providers can balance the need for rapid diagnosis with the emotional well-being of patients.

This duality is further observed by Cornford et al. [17], who demonstrated that while patients value the fast investigation process, they often feel they lack understanding of why it needs to happen so quickly. This emphasizes the importance of clear and proactive communication from healthcare providers to better address patients’ anxiety and expectations. Our findings were also align with results by Ndukwe et al. [18] who showed that patients referred according to SCP typically appreciated the swift referral and investigation process, but experienced significant anxiety and distress due to both their symptoms and the referral procedure.

Informants frequently linked the urgency to the possibility of a serious medical condition, which increased their anxiety even before the hospital visit. The standardized care pathway aimed to early detect malignancies such as UBC which ranked as one of the costliest cancers per patient, mainly due to the need for prolonged follow-up and the high risk of recurrence [19]. However, the positive effects of SCP on waiting times, care efficiency, and patient flow were highlighted by Borg et al. [20], and successfully reduced waiting times for both diagnosis and treatment. By standardizing and streamlining care processes, it enables patients to undergo essential tests and treatments without unnecessary delays. This has led to more consistent and quicker treatment results, potentially improving long-term survival for cancer patients.

The emotional toll during the diagnostic process was clearly evident in the informants’ stories. While the standardized care pathway helped reduce long-term uncertainty, it also intensified fears of serious diagnoses. For some, stress increased due to undergoing tests and receiving immediate results without sufficient preparation. Despite this, many informants found reassurance in the timely handling of investigations, which helped ease their anxiety and uncertainty.

Informants voiced frustration about receiving unexpected calls from the urologist, particularly when these calls occurred in inconvenient situations. The absence of additional communication, such as digital messages, left patients feeling unprepared and uncertain about the next steps. Our results show that the absence of a clear explanation about why their cases were being investigated so quickly caused anxiety and confusion for many informants. They believed that receiving more information about the reasons for the fast referral to a specialist would have eased their concerns about the speed of the process. This highlights the psychological effects of a fast-track system and emphasizes the need for improved communication and support to reduce unnecessary worry. This was in accordance with findings from other studies that highlight communication as a key area for improving patient experience in UBC diagnostics [21].

A recurring topic in the interviews was the variation in the clarity and sufficiency of the information provided to patients by both the primary care centre and the phone call from the urological unit. While many appreciated explanations during their hospital visits, others felt inadequately informed about the reasons behind the fast-track investigation or the possible outcomes of the tests. This inconsistency emphasizes the need to tailor communication to individual patient needs and ensure that all patients receive clear and consistent information. Additionally, patients expressed a preference for written communication, such as SMS or email, to better manage and comprehend the information. This insight is essential for enhancing patient communication and ensuring patients are well-informed.

Continuing with a one-stop clinic policy, where all procedures are completed on the same day, proves to be highly beneficial. This approach streamlines the diagnostic process, minimizing the need for multiple visits and reducing the overall waiting time for patients. It enhances convenience and helps alleviate the stress and uncertainty associated with prolonged delays between appointments. Supporting this, other studies have demonstrated that one-stop policy for investigation of macroscopic hematuria effectively streamline the diagnosis of urological malignancies [22]. These findings underscore the value of such setting up in facilitating early and accessible urological care.

**Fig. 2:**
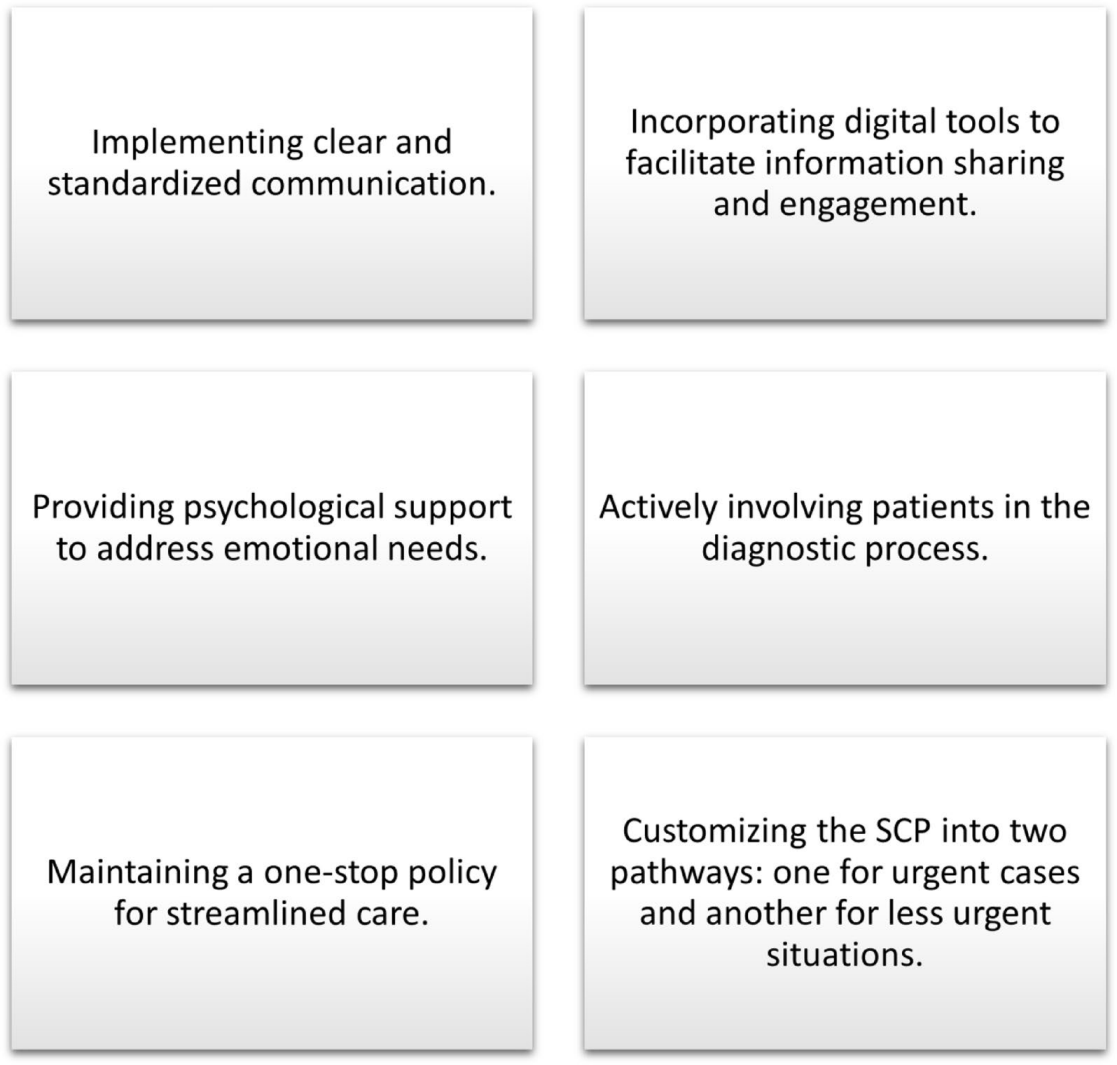
Several strategies could enhance patients’ experiences within the Standardized care pathway

The findings suggest several strategies that could improve patients’ experiences within SCP (Fig. 2). Clear information and individualised communication are essential, as explaining the reasons for urgency and potential outcomes of tests can help alleviate anxiety. The use of digital tools to provide practical and preparatory information could enhance patients’ understanding and satisfaction. Psychological support is also important; offering brief consultations or informational materials before invasive procedures can make patients feel more prepared and less anxious. Involving patients in the diagnostic process, such as allowing them to view images from cystoscopies or X-rays, can create a sense of control and engagement.

One of the strengths of our study is that the interviews were conducted shortly after the diagnostic investigations, allowing us to capture a clear and accurate picture of patients’ experiences with the SCP. Moreover, the use of semi-structured interviews in this study provides rich, in-depth insights into patients’ subjective experiences with SCP for macroscopic hematuria. Furthermore, by interviewing 12 patients, the study captures a diverse range of individual experiences, offering valuable perspectives on how different patients perceive and navigate the SCP.

However, the study has certain limitations. The semi-structured interview format, while flexible, is subjective and relies on patients’ personal interpretations of their experiences, which could lead to biases in how they recount or frame their stories. Furthermore, the limited diversity of the sample—consisting mostly of patients who were not diagnosed with UBC, with only one confirmed cancer case—may restrict the breadth and depth of the findings. This imbalance could limit the ability to capture the full range of experiences, particularly those unique to patients who receive a cancer diagnosis. Additionally, knowing the diagnostic outcome prior to the interview may have influenced patients’ reflections and perceptions of navigating the SCP, potentially affecting how they recall and interpret their experiences. These factors should be considered when interpreting and generalizing the results.

## Conclusions

This study highlights the complex emotional landscape patients navigate during the diagnostic process for macroscopic hematuria. While rapid diagnostic workup through SCP can reduce waiting times, it does not automatically alleviate anxiety, especially when information is unclear or lacking. Our findings underscore the importance of clear and empathetic communication throughout the diagnostic pathway—both in primary care and at the specialist level.

Although the one-stop clinic model was appreciated by some patients for its efficiency, others found the process rushed or emotionally overwhelming, particularly when they felt unprepared. These differences suggest a need not necessarily for individually tailored protocols, but rather for more consistent and thorough communication and support across care providers. Our single-center findings may be applicable to similar healthcare systems seeking to streamline cancer diagnostics. Future studies could investigate how to integrate preparatory discussions and digital tools to better support patients in fast-tracked diagnostic pathways.

## Supplementary Information

Below is the link to the electronic supplementary material.

Supplementary Material 1

Supplementary Material 2

Supplementary Material 3

## Data Availability

The datasets used and/or analysed during the current study are available from the corresponding author on reasonable request.

## Abbreviations

CTU: Computed tomography urography
QoL: Quality of life
SCP: Standardized care pathway
UBC: Urinary bladder cancer

## Declarations

### Clinical trial number

Clinical trial number: not applicable.
