# Supplementary material for: Patients’ experiences of a standardized care pathway for suspected bladder cancer due to macroscopic hematuria
Source: BMC Urol. 2025 Aug 23;25:216. doi: 10.1186/s12894-025-01898-1 (PMC12374357; doi:10.1186/s12894-025-01898-1)
Supplement: Supplementary file 1 — Supplementary Material 1 [file 12894_2025_1898_MOESM1_ESM.docx]

# Patients’ experiences of a standardized care pathway for suspected bladder cancer due to macroscopic hematuria

Lisa Karlsson^1^, Anette Ek-Steinum^1^, Viola Nyman^2,3,4,^ and Suleiman Abuhasanein^1,4,5^

# Consolidated criteria for reporting qualitative studies (COREQ): 32-item checklist

| **N.** | **Item + Guide questions/description** | |
| --- | --- | --- |
| **Domain 1: Research team and reflexivity** | | |
| **Personal Characteristics** | | |
| 1 | Interviewer/facilitator Which author/s conducted the interview or focus group? | AE |
| 2 | Credentials What were the researcher’s credentials? E.g. PhD, MD | LK and AE Registered Nurse  VN is Associate Professor, Registered Nurse and Registered Midwife  SA is consultant/urologist, MD. PhD. FEBU. |
| 3 | Occupation What was their occupation at the time of the study? | Clinical nurse |
| 4 | Gender Was the researcher male or female? | Female and male |
| 5 | Experience and training What experience or training did the researcher have? | All authors are accustomed to interviewing |
| **Relationship with participants** | | |
| 6 | Relationship established Was a relationship established prior to study commencement? | AE and SA work at the same clinic where the patients had attended for investigation of macroscopic hematuria and may have encountered some of them previously in their routine clinical roles. |
| 7 | Participant knowledge of the interviewer. What did the participants know about the researcher? e.g. personal goals, reasons for doing the research | Participants had no prior personal acquaintance with the researchers |
| 8 | Interviewer characteristics What characteristics were reported about the interviewer/facilitator? e.g. Bias, assumptions, reasons and interests in the research topic | The interviewer (AE) is experienced nurses with clinical expertise in urology. She had a professional interest in improving patient care and communication. Her potential biases and assumptions were discussed within the research team to promote reflexivity. |
| **Domain 2: study design** | | |
| **Theoretical framework** | | |
| 9 | Methodological orientation and Theory What methodological orientation was stated to underpin the study? e.g. grounded theory, discourse analysis, ethnography, phenomenology, content analysis Participant selection | Content analysis by Graneheim and Lundman |
| 10 | Sampling How were participants selected? e.g. purposive, convenience, consecutive, snowball | Participants were selected using a combination of purposive and convenience sampling. |
| 11 | Method of approach How were participants approached? e.g. face-to-face, telephone, mail, email | Face to face. |
| 12 | Sample size How many participants were in the study? | 12 |
| 13 | Non-participation How many people refused to participate or dropped out? Reasons? | Six patients declined to participate. Reasons included lack of time, not feeling well enough, or not wanting to talk about their experience. |
| **Setting** | | |
| 14 | Setting of data collection Where was the data collected? e.g. home, clinic, workplace | Participants were selected from those attending the outpatient clinic for investigation of macroscopic hematuria between April and November 2023. |
| 15 | Presence of non-participants Was anyone else present besides the participants and researchers? | No, only the participants and the researchers were present during the interviews. |
| 16 | Description of sample What are the important characteristics of the sample? e.g. demographic data, date | The final sample included 12 informants, of whom 7 (58%) were women, with a median age of 71 years (IQR 65–76). One participant was diagnosed with urothelial bladder cancer (UBC), while the remaining had benign causes for their macroscopic hematuria. |
| **Data collection** | | |
| 17 | Interview guide Were questions, prompts, guides provided by the authors? Was it pilot tested? | An interview guide was used including a main opening question:  "Can you start by describing the first time you noticed blood in your urine?"  There were follow-up questions if needed. Please see suppl 2. |
| 18 | Repeat interviews Were repeat interviews carried out? If yes, how many? | No |
| 19 | Audio/visual recording Did the research use audio or visual recording to collect the data? | Audio recording was used. |
| 20 | Field notes Were field notes made during and/or after the interview or focus group? | No |
| 21 | Duration What was the duration of the interviews or focus group? | Approximately 40-50 minutes each. |
| 22 | Data saturation Was data saturation discussed? | Yes, data saturation was reached when no new information regarding the standardized care pathway (SCP) emerged during the interviews. |
| 23 | Transcripts returned Were transcripts returned to participants for comment and/or correction? | No |
| **Domain 3: analysis and findings** | | |
| **Data analysis** | | |
| 24 | Number of data coders How many data coders coded the data? | LK and SA |
| 25 | Description of the coding tree Did authors provide a description of the coding tree? | Yes, the authors provided a detailed description of the coding tree in a table format, outlining main themes, subthemes, and corresponding codes used during data analysis. |
| 26 | Derivation of themes Were themes identified in advance or derived from the data? | Themes were derived from the data. |
| 27 | Software What software, if applicable, was used to manage the data? | No software was used during the analysis. |
| 28 | Participant checking Did participants provide feedback on the findings? | No, but they can receive the paper if they had wanted? |
| **Reporting** | | |
| 29 | Quotations presented Were participant quotations presented to illustrate the themes / findings? Was each quotation identified? e.g. participant number | Quotations were used and identified with an informant number. |
| 30 | Data and findings consistent Was there consistency between the data presented and the findings? | Efforts were made to maintain transparency between the interview quotes and the data interpretation. |
| 31 | Clarity of major themes Were major themes clearly presented in the findings? | Yes |
| 32 | Clarity of minor themes Is there a description of diverse cases or discussion of minor themes? | There were no minor themes presented. |
