# Supplementary material for: Patients’ experiences of a standardized care pathway for suspected bladder cancer due to macroscopic hematuria
Source: BMC Urol. 2025 Aug 23;25:216. doi: 10.1186/s12894-025-01898-1 (PMC12374357; doi:10.1186/s12894-025-01898-1)
Supplement: Supplementary file 2 — Supplementary Material 2 [file 12894_2025_1898_MOESM2_ESM.docx]

# The interview guide

**Study: Patients’ experiences of a standardized care pathway for suspected bladder cancer due to macroscopic hematuria**

**Introduction to the Interview:**

Thank you for taking the time to participate in this interview. We are interested in learning about your experiences from the time you first noticed blood in your urine, through your contact with healthcare, and any diagnostic or treatment processes you may have gone through. There are no right or wrong answers—we want to understand your perspective. Your responses will help improve care for future patients.

**Main Opening Question:**

"Can you start by describing the first time you noticed blood in your urine?"

**Follow-up and Probing Questions (as needed):**

1. How did you react when you saw the blood?

2. What did you do after that? Did you seek care immediately or wait?

3. Can you describe your first contact with healthcare regarding this issue?

4. How were you informed about the care pathway or the next steps?

5. How did you experience the communication with healthcare professionals?

6. Were the investigations or procedures explained to you clearly?

7. How did you feel emotionally during the process?

8. Did you feel supported throughout your journey?

9. Were there any moments you found particularly stressful, reassuring, or confusing?

10. Looking back, is there anything you think could have been done differently or better?

**Closing Question:**

"Is there anything else you would like to share about your experience that we haven’t talked about?"
