# Supplementary material for: Patients’ experiences of a standardized care pathway for suspected bladder cancer due to macroscopic hematuria
Source: BMC Urol. 2025 Aug 23;25:216. doi: 10.1186/s12894-025-01898-1 (PMC12374357; doi:10.1186/s12894-025-01898-1)
Supplement: Supplementary file 3 — Supplementary Material 3 [file 12894_2025_1898_MOESM3_ESM.pdf]

Patienters upplevelse av diagnosprocessen enligt standardiserat vårdförlopp för makroskopisk hematuri.
En kvalitativ intervjustudie.

2022-01055-01
Grundansökan
Grundansökan
Avslutad

Suleiman Abuhasanein

1.2 Ansvarig huvudman för forskningen (forskningshuvudman)

Västra Götalandsregionen (232100-0131)

1.3 Behörig företrädare för forskningshuvudman

Maria Wiksten Ericsson

1.3.1 Behörig företrädare – titel som innebär ett verksamhetsansvar

Verksamhetschef

1.4 Har projektet fler forskningshuvudmän?

Nej

1.5 Hemvist för forskningen

Urologsektionen, Kirurgkliniken NU sjukvården

1.6 Huvudansvarig forskare för projektet (kontaktperson)

Suleiman Abuhasanein

1.6.1 Institution/hemvist som huvudansvarig forskare är verksam vid

Department of Urology, Institute of Clinical Science, Sahlgrenska Academy, University of Gothenburg

1.7 Är den huvudansvariga forskaren disputerad?

Nej

1.7.1 [Om Nej 1.7] Ange den [disputerade] forskare som kommer utöva att aktivt överinseende över forskningen

Viola Lindberg Nyman

1.7.2 [Om Nej 1.7] Ange den [disputerade] forskarens titel

Barnmorska/Midwife PhD, Forskningsledare/Research director Department of Research and Development NU-Hospital Group. Mail viola.nyman@vgregion.se

Frågor för avgiftskategori

1.9 Hur många forskningshuvudmän kommer att ingå i forskningsprojektet?

En

1.10 Avser forskningen klinisk läkemedelsprövning?

Nej

1.11 Ska endast befintliga personuppgifter behandlas i projektet?

Nej

2.1 Avser ansökan forskning som inbegriper äggdonation?

Nej

2.2 Avser ansökan forskning med läkemedel för genterapi eller somatisk cellterapi eller läkemedel som innehåller genetiskt modifierade organismer?

Nej

2.3 Avser ansökan forskning med xenogen cellterapi?

Nej

2.4 Kommer joniserande strålning ingå i forskningsprojektet?

Nej

2.5 Kommer biologiskt material från människor att nyinsamlas för projektet?

Nej

2.6 Planerar projektet att använda biologiskt material från människor från en eller flera befintliga provsamlingar?

Nej

2.7 Avser forskningen klinisk prövning eller en prestandastudie av medicinteknisk produkt/medicinteknisk produkt för in vitro-diagnostik?

Nej

2.8 Gör en egen bedömning och ange på vilka punkter nedan som forskningen omfattas av 3-4 §§ etikprövningslagen. Observera att myndigheten kan komma att göra en annan bedömning.

✓ 3 § 1 Forskningen kommer att samla in känsliga personuppgifter.

2.8.1 [Om 3 § 1] Gör en egen bedömning och ange vilken typ av känsliga personuppgifter som kommer att behandlas i projektet. Observera att myndigheten kan komma att göra en annan bedömning.

✓ hälsa

2.9 Önskas ett rådgivande yttrande?

Ja

2.10 Söker projektet förtur med motivering att projektet har tydlig potential att ge nytta i närtid för behandling och förebyggande av COVID-19?

Nej

3.1 Skriv en populärvetenskaplig sammanfattning av forskningsprojektet.

Urinblåsecancer (UBC) är den 7:e vanligaste cancerformen hos män i världen, medan den är den 11:e när båda könen beaktas. De största riskfaktorerna för BC är rökning, och hög ålder.
Makroskopisk hematuri (blod i urinen) är det vanligaste symtomet för UBC. Generellt sett kan en cancerdiagnosfördröjning ha en negativ inverkan på prognosen. Därför implementerades i Sverige under 2016 ett standardiserat vårdförlopp (SVF) som ett snabbspår för patienter med misstänkt UBC, främst på grund av makroskopisk hematuri. Ambitionen var att patienter (50 år eller äldre) ska uppleva en välorganiserad och professionell vård, oavsett var i landet patienter söker vård, samt för att minska ledtider. SVF består av en enhetlig utredning inklusive cystoskopi och skiktröntgen (computed tomography). För att minska tiden från första symtom till behandling var avsedd ledtid för utredning från remiss till operation 13 dagar.
Handläggning av makroskopisk hematuri med att ha i åtanke att man kan ha UBC är naturligtvis en stressande process. Att patienter får vänta länge för att komma till specialiserad vård är ett känt fenomen inom den svenska sjukvården, vilket gör det ännu viktigare att undersöka påverkan av så snabb handläggning på patienternas mående och ifall de upplever situationen som onödigt stressigt. Även om livskvaliteten (QoL) för UBC-patienter har beskrivits väl i litteraturen, saknas fortfarande en fullständig beskrivning av patienternas upplevelser under diagnosprocessen. Såvitt vi vet har inga intervjubaserade studier utförts för individer med MH som genomgår en snabb diagnosprocess som SVF.

3.2 Vad är det vetenskapliga syftet med projektet?

Denna intervjustudie syftar till
1-att beskriva hur patienter med makroskopiskt hematuri upplever det standardiserade vårdförloppet för UBC inklusive cystoskopi.

2-att beskriva hur patienter uppfattar det diagnostiska ingreppet (transuretral resektion av urinblåsan-TURB).

3.3 Vilka är de vetenskapliga frågeställningarna?

1-Hur uppfattar patienter med MH mediciniska och professionella hantering och mottagandet under SVF?
2-Hur påverkar den snabba handläggningen patienternas mående?
3-Vad är patienternas upplevelser under cystoskopi på mottagningen?
4-Hur upplever patienter diagnostiska ingreppet (transuretrala resektionen av urinblåsan TURB)?

4.1 Redogör för metod inkl. proceduren, tekniken eller behandlingen.

En intervjustudie planeras för att utforska patienters upplevelse av SVF-process för makroskopisk hematuri. Patienter kommer att bli kontaktade efter att de genomgick utredningen, man frågar de ifall de vill delta i studien. Denna studie kommer att baseras på individuella intervjuer med en inledande öppen fråga: Kan du börja berätta från att du första gången fick se blod i urinen? Sedan får patienter berätta fritt om sina upplevelser av vård hantering av deras ärenden. Intervjuer ska genomföras antingen fysiskt, via telefon eller videosamtal och varar i cirka 35–45. Intervjuer kommer att spelas in och transkriberas därefter ordagrant.
Data kommer att analyseras genom innehållsanalys med induktiv ansats enligt Lundman & Graneheim. Denna metod är en relevant metod i kvalitativa studier där både det manifesta och latenta budskapet kan belysas och fokus ligger på att beskriva variationer i texten och identifiera mönster. Enligt Gillham är en intervju en vedertagen metod för att få en direkt insyn av informanters upplevelser, känslor och tankar av det studerade fenomenet (den snabba processen av diagnostisering av eventuell cancer efter att patienter ser blod i urinen). Ett krav för en lyckad induktiv ansats är att materialet skall bearbetas förutsättningslöst så att informanternas upplevelser hamnar i fokus.

4.2 Redogör för på vilket sätt metoden skiljer sig från klinisk rutin eller den ordinarie behandlingen.

Vanligtvis kontaktar patienter primärvården efter att man ser blod i urinen, primärvården skickar en remiss till urologen som gör en individuell bedömning. I de flesta fall gör man en cystoskopi inom 2-4 veckor. Under SVF ska patienter utredas inom viss tidsram (inom 6 dagar). Det är svårt för vissa patienter att förstå varför det är så bråttom (med tanke på att cancerrisk i denna kohort är runt 15%), d.v.s. majoriteten utreds dock ingen cancerdiagnos.

4.3 Redogör för tidigare erfarenheter (egna och/eller andras) av den använda proceduren, tekniken eller behandlingen.

Inblandade forskare har erfarenhet av utredning och behandling av urinblåsecancer patienter.
Viola Nyman har erfarenhet av intervjustudier.
Dessutom är kvalitativa metoder väl etablerade inom vetenskapliga forskningen och kan fånga information som finns ”mellan” frågorna, t ex i ett skattningsformulär och ge en ytterligare dimension till den samlade kunskapen om hur människor upplever olika situationer i sitt liv.

5.1 Förväntat startdatum för projektet:

sommar 2022

5.2 Förväntat slutdatum för projektet:

sommar 2024

5.3 Tidsplan för de olika delar som ingår i projektet:

Intervjuer kommer att börja spelas in sommaren/hösten 2022. 0.5-1 år behövs för att analysera och skriva rapport.

6.1 Redogör för datainsamling och datas karaktär.

Semi strukturerad intervjuer samt en kompletterande retrospektiv journalgenomgång för att söka relevanta information.

6.2 Redogör för det statistiska underlaget för studiepopulationen/ undersökningsmaterialets storlek.

Studien skall ha cirka 15 deltagare.

6.3 Hur kommer undersökningsprocedurerna att dokumenteras?

Dokumentationen kommer att innehålla bandinspelning som ska transkriberas inför analys.

6.4 Hur kommer insamlad data att hanteras och förvaras?

Samtliga kliniska variabler kommer att förvaras lösenordskyddat i databas på säker server hos huvudmannen. Endast behöriga forskare kommer att ha tillgång till studiedata. Studiedata kommer att sparas i 10 år och arkiveras i enlighet med regionens rutiner.

7.1 Vilka risker kan ett deltagande medföra för de forskningspersoner som ingår i forskningsprojektet?

Studien innebär ett intrång i den personliga integriteten, då vi inhämtar data från patientjournaler, men all kliniska data kommer att pseudonymiseras så att det inte går att känna igen patienter efter analysering av data.

7.2 Vilken nytta kan ett deltagande medföra för de forskningspersoner som ingår i forskningsprojektet?

inget

7.3 Gör en värdering av förhållandet mellan riskerna och nyttan av projektet.

Nyttan med studien bedöms överstiga risken, då den syftar till att öka förståelse av patienters upplevelse av den mediciniska hanteringen i samband med utredning av MH under SVF.

7.4 Beskriv hur projektet har utformats för att minimera riskerna för forskningspersonerna.

Denna studie är en kvalitativ intervjustudie. Inklusionen börjar efter att patienterna genomgick den utredning som vanligtvis görs för alla patienter med makroskopisk hematuri, vilket gör att de löper inga risker som kan påverka deras utredning som är redan avklarad.
De patienterna som fick cancerdiagnos skall fortsatt behandlas i kliniken på ett sedvanligt sätt. Deras tankar och upplevelser kommer att hjälpa till att förstå mer och korrigera eventuella svagheter i hanteringen snarare än att riskera deras chans att få den behandling som behövs. Anonymitet av all data såvida att det inte går att koppla ihop data med en enskild patient kommer att minimera riskerna för forskningspersonerna.

7.5 Identifiera och precisera om eventuella etiska problem (nackdelar/fördelar) kan uppstå i ett vidare perspektiv genom forskningsprojektet.

En eventuell risk är att forskningspersonerna namnger vissa vårdpersonal och pekar ut dem som oprofessionella vilket kan möjligen påverka dem negativt. Genom anonymisering av all data kommer den risken att minimeras.

8.1 Hur görs urvalet av forskningspersoner?

Patienter som genomgår SVF för MH kommer att frågas vid kontakten på mott om de vill ingå i studien.

8.2 Hur många forskningspersoner kommer att inkluderas i forskningsprojektet?

Studien skall inkludera cirka 15 deltagare.

8.3 Vilka urvalskriterier kommer att användas för inklusion?

Deltagarna ska vara 50 år eller äldre, och ha genomgått SVF för MH.

8.4 Vilka urvalskriterier kommer att användas för exklusion?

Ålder<50 år

8.5 Ange relationen mellan forskare och forskningspersonerna.

Patienter sökte redan vård och det kan hända att undertecknad eller en av forskarna som jobbar med projektet har träffat en av patienterna, men detta kommer naturligtvis inte påverka själva arbetet.

8.6 Vilket försäkringsskydd finns för de forskningspersoner som deltar i forskningsprojektet?

Patienter är försäkrade genom Patientförsäkringen.

8.7 Redogör för den beredskap som finns för att hantera oväntade bifynd eller händelser under forskningsprocessen som kan äventyra forskningspersonernas säkerhet.

ej aktuellt.

8.8 Kommer ekonomisk ersättning eller andra förmåner betalas ut till forskningspersonerna?

Nej

9.1 Kommer forskningspersonerna att informeras om forskningsprojektet och tillfrågas om de vill vara med eller inte?

Ja

9.1.1 [Om Ja 9.1] Hur, när (i vilket skede) och av vem informeras och tillfrågas forskningspersonerna?

Patienterna kommer att kontaktas inom ck 30 dagar efter avslutad utredning enligt SVF för MH och de kommer att ges möjligheten att förstå innehållet av studien och vad denna innebär och ges möjlighet att fråga och tänka om de vill vara med och i så fall få lämna ett skriftligt samtycket.

9.2 Kommer barn under 18 år att ingå i forskningsprojektet?

Nej

9.3 Kommer forskningspersoner, vars mening på grund av sjukdom, psykisk störning, försvagat hälsotillstånd eller något annat liknande förhållande inte kan inhämtas, att ingå i forskningsprojektet?

Nej

10.1 Kommer projektet att begära ut uppgifter från ett befintligt register?

Ja

10.1.1 [Om Ja 10.1] Ur vilket eller vilka register kommer uppgifterna att begäras?

Journaldata

10.1.2 [Om Ja 10.1] Vilka uppgifter kommer att begäras ut och varför?

Primär Diagnos
Ålder
kön
Urologiska symtom

Resultat på röntgen utlåtande
Resultat på cystoskopi
resultat av TURB
PAD efter TURB

11.1 Finns det relevanta resultat från djurförsök?

Ej aktuellt

12.1 Hur garanteras tillgång till data för forskningshuvudmannen och medverkande forskare?

Samtliga medverkande forskare kommer att ha tillgång till data.

12.2 Vem eller vilka ansvarar för databearbetning och skriftlig redovisning av resultaten?

Samtliga medarbetare ansvarar gemensamt för analys och redovisning.

12.3 Hur och när planeras resultaten att offentliggöras?

Studien kommer att publiceras i internationella tidskrifter med peer-review, samt vid nationella och internationella möten.

12.4 På vilket sätt garanteras forskningspersonernas rätt till integritet när materialet offentliggörs?

En särskild omsorg kommer att vidtas för att inga individer kan identifieras.

13.1 Redovisa eventuella ekonomiska överenskommelser med bidragsgivare eller andra finansiärer (namn och belopp).

Studien finansieras genom forskningsmedel från Västra Götalandsregionen.

13.2 Redovisa forskningshuvudmannens, huvudansvarig forskares och medverkande forskares egna ekonomiska intressen.

Inga ekonomiska intressen föreligger

Forskningsplan

Den sammanfattande beskrivningen av forskningsprojektet ska förstås av fackmän. Den kan lämpligen utformas enligt följande:
Vetenskaplig frågeställning: En redogörelse för det övergripande syftet med det föreslagna forskningsprojektet samt specifika mål (primära och sekundära frågeställningar).
Områdesöversikt: Ge ett sammandrag av egna och andras forskning och tidigare resultat inom forskningsområdet. Översikten ska tydliggöra det aktuella projektets relevans. Nyckelreferenser ska anges.
Projektbeskrivning: Gör en sammanfattning av projektets/motsvarande uppläggning. Urval av

forskningspersoner, procedurer, metoder med mera ska tydligt redovisas. Det ska framgå hur metoder, urval och procedurer kan ge svar på de specifika frågeställningarna. Om flera delprojekt avses anges sekvens för genomförande och på vilket sätt ett efterföljande delprojekts uppläggning kan bero av resultaten av ett föregående.
Betydelse: Ge en kortfattad redogörelse för projektets betydelse för forskningsområdet.
Preliminära resultat: Kan i förekommande fall anges.
SKA VARA PÅ SVENSKA ELLER ENGELSKA.

Kv.s._Projektplan.docx.pdf
118.45KB

Kv.s._Projektplan_edited.docx.pdf
95.72KB

Information till forskningspersoner och samtyckesformulär

Etikprövningsmyndigheten behöver alltid ta del av all information som kommer att ges till forskningspersonen i samband med tillfrågan om deltagande. Både den information som ska ges muntligt och den som ska ges skriftligt. Om vårdnadshavare ska samtycka till deltagande ska även den information som ges till vårdnadshavarna bifogas. Om anhörig ska ges möjlighet att motsätta sig deltagande ska även den information som ges till anhörig bifogas.
Etikprövningsmyndigheten rekommenderar att vår stödmall för forskningspersonsinformation används, den hittar du på www.etikprovning.se
SKA VARA PÅ SVENSKA.

bilaga_1_kva_samtyckefrmulret.docx.pdf
80.44KB

bilaga_0_kva_studiebalnket.docx.pdf
78.1KB

bilaga_0_studiebalnket_ny_version.docx.pdf
79.45KB

Enkäter, frågeformulär, mm

Materialet ska vara utformat/skrivet på svenska.
SKA VARA PÅ SVENSKA.

Intervjuguide.docx.pdf
66.92KB

CV för ansvarig forskare

Bifoga CV för ansvarig forskare.
I undantagsfall kan icke disputerad forskare godtas om annan medverkande disputerad forskare uttalat att forskningen sker under aktivt överinseende av denne. Uttalandet intygas vid signering

av disputerad forskare. CV för den disputerade ska även bifogas.
SKA VARA PÅ SVENSKA ELLER ENGELSKA.

CV-Suleiman.pdf
735.9KB

CV_Viola_Nyman_220127.pdf
198.06KB

I och med att ansökan undertecknas intygar du som är ansvarig forskare samt du som är behörig företrädare följande;

- Att den information som lämnas i ansökan om etikprövning och samtliga medföljande bilagor är riktig och fullständig.
- Att verksamhetsansvariga i samtliga medverkande verksamheter är informerade om forskningsprojektets innehåll och utförande och att de har samtyckt till att delta i studien.
- Att du säkerställt att det i samtliga medverkande verksamheter finns resurser som garanterar forskningspersonernas säkerhet och integritet vid genomförandet av den forskning som beskrivs i ansökan.
- Att ansvarig forskare ges rätt att företräda huvudmannen i alla framtida kontakter med Etikprövningsmyndigheten som rör detta forskningsprojekt samt ansöka om ändringar i forskningsprojektet.
- Att du tagit del av Etikprövningsmyndighetens information om hantering av personuppgifter på myndighetens webbplats.

Behörig företrädare för forskningshuvudmannen

Maria Viksten Ericsson

Behörig företrädare saknar Bank-id

✓ Om den behörige företrädaren saknar Bank-id, klicka här.

Signatur behörig företrädare

Signatur-behorig-foretradare.pdf
31.83KB

Är behörig företrädare ordinarie eller tillförordnad?

Ordinarie företrädare

Huvudansvarig forskare saknar Bank-id

✓ Om den huvudansvarige forskaren saknar Bank-id, klicka här.

Signatur huvudansvarig forskare

Signatur-huvudansvarig-forskare.pdf

30.15KB

Signatur-huvudansvarig-forskare.pdf
31.03KB

Signatur-huvudansvarig-forskare.pdf
31.22KB

Signatur-huvudansvarig-forskare.pdf
31.33KB

Disputerad forskare som kommer utöva att aktivt överinseende över forskningen. Ska överensstämma med fråga 1.7.1.

Viola Maria Katariina Nyman

Signatur disputerad forskare

Signatur-disputerad-forskare.pdf
27.55KB

Svar på de frågor eller synpunkter som Etikprövningsmyndigheten angett i sitt beslut, samt beskrivning av de ändringar som gjorts

Tack så mycket för era punkter, härnedan är svar på dem

1. Intervjuguide ska bifogas ansökan...... den är bifogad nu.
2. Kommer även SVF-UBC-patienter där utredningen varit negativ, dvs ingen urotelial cancerpåvisats, att kunna inkluderas i studien? .... Inte i denna studie, ifall de ska inkluderas behöver vi komplettera ansökan?
3. I forskningspersoninformationen ska syftet med studien beskrivas tydligare (det är tydligarebeskrivet i ansökans punkt 3.2)............ den är tydliggjord nu.
4. I forskningspersoninformationen ska:
a. tillfogas information om att "Studien är godkänd av Etikprövningsmyndigheten, dnr 2022-01055-01"...........den är tillagd nu.
b. "Datainspektionen" ändras till "Integritetsskyddsmyndigheten (www.imy.se)"..............den är ändrad nu.

Tack igen och trevlig sommar!
/Suleiman

Beslut och handlingar från Etikprövningsmyndigheten

Beslutsbrev och andra handlingar från Etikprövningsmyndigheten i relation till denna ansökan

2022-01055-01_Avgiftsavisering.pdf
35.3KB

2022-01055-01_Begaran_om_administrativt_tillagg.pdf
41.1KB

2022-01055-01_Ansokan_Komplettering_till_sammantrade.pdf
46.39KB

2022-01055-01_Ansokan_komplettering_till_ordforande.pdf
43.76KB

2022-01055-01_Ansokan_Godkand.pdf
37.57KB

registrator@etikprovning.se | 010-475 08 00 | Box 2110, 750 02 Uppsala | **etikprovning.se**

Patienters upplevelse av diagnosprocessen enligt standardiserat vårdförlopp för makroskopisk hematuri

En kvalitativ intervjustudie

Projektplan

Etikprövningsmyndigheten
2022-01055-01-229278
2022-02-23

Bakgrund

Urinblåsecancer (UBC) är den 7:e vanligaste cancerformen hos män i världen, medan den är den 11:e när båda könen beaktas [1]. De största riskfaktorerna för BC är rökning, och hög ålder [2]. Makroskopisk hematuri (blod i urinen) är det vanligaste symtomet för UBC [3]. Generellt sett kan en cancerdiagnosfördröjning ha en negativ inverkan på prognosen [4]. Därför implementerades i Sverige under 2016 ett standardiserat vårdförlopp (SVF) som ett snabbspår för patienter med misstänkt UBC, främst på grund av makroskopisk hematuri. Ambitionen var att patienter (50 år eller äldre) ska uppleva en välorganiserad och professionell vård, oavsett var i landet patienter söker vård, samt för att minska ledtider [5]. SVF består av en enhetlig utredning inklusive cystoskopi och skiktröntgen (computed tomography) [5]. För att minska tiden från första symtom till behandling var avsedd ledtid för utredning från remiss är 13 dagar.

Handläggning av makroskopisk hematuri med att ha i åtanke att man kan ha UBC är naturligtvis en stressande process [6]. Att patienter får vänta länge för att komma till specialiserad vård är ett känt fenomen inom den svenska sjukvården, vilket gör det ännu viktigare att undersöka påverkan av så snabb handläggning på patienternas mående och ifall de upplever situationen som onödigt stressigt. National Comprehensive Cancer Network (NCCN) definierar stress som

> "en multifaktoriell obehaglig upplevelse av psykologisk (dvs. kognitiv, beteendemässig, emotionell), social, andlig och/eller fysisk natur som kan störa förmågan att hantera cancersymtom och dess behandling" [7].

Även om livskvaliteten (QoL) för UBC-patienter har beskrivits väl i litteraturen, saknas fortfarande en fullständig beskrivning av patienternas upplevelser under diagnosprocessen [8-10]. Att utföra kvalitetsbedömningar i klinisk praxis har visat sig inte bara öka vårdgivarens och patientens medvetenhet om behandlingsrelaterade frågor, utan kan förbättra kommunikationen

Etikprövningsmyndigheten
2022-01055-01-229278
2022-02-23

mellan patienter och vårdpersonal, samt förbättra patienternas överlevnad [11]. Såvitt vi vet har inga intervjubaserade studier utförts för individer med MH som genomgår en snabb diagnosprocess som SVF.

Syfte

Denna intervjustudie syftar till att beskriva hur patienter med makroskopiskt hematuri upplever det standardiserade vårdförloppet för diagnosticering av UBC samt beskriva hur patienter uppfattar det diagnostiska ingreppet (transuretral resektion av urinblåsan-TURB) under spinal anestesi (ryggbedövning).

Metod

Etikprövningsmyndigheten
2022-01055-01-229278
2022-02-23

En intervjustudie planeras för att utforska patienters upplevelse av SVF för makroskopisk hematuri. I resten av detta dokument kommer patienter att kallas informanter. Data kommer att analyseras genom innehållsanalys med induktiv ansats enligt Lundman & Graneheim [12]. Denna metod är en relevant metod i kvalitativa studier där både det manifesta och latenta budskapet kan belysas och fokus ligger på att beskriva variationer i texten och identifiera mönster [12]. Enligt Gillham är en intervju en vedertagen metod för att få en direkt insyn av informanters upplevelser, känslor och tankar av det studerade fenomenet (den snabba processen av diagnostisering av eventuell cancer efter att patienter ser blod i urinen) [13].

Denna studie kommer att baseras på individuella intervjuer med en inledande öppen fråga: *Kan du börja berätta från att du första gången fick blod i urinen*? Ett krav för en lyckad induktiv ansats är att materialet skall bearbetas förutsättningslöst så att informanternas upplevelser hamnar i fokus. Intervjuer ska genomföras via telefon eller videosamtal på grund av restriktioner

gällande Covid-19 och varar i cirka 35–45. Intervjuer kommer att spelas in och transkriberas därefter ordagrant.

Inklusionskriterier

Deltagarna ska vara 50 år eller äldre, ha tillräckliga kunskaper i svenska för att kunna delta i en intervju och ha genomgått SVF för makroskopisk hematuri inom 60 dagar så att diagnosprocessen finns färskt i minnet.

Datainsamling

Första studien: Patienters upplevelser av diagnosprocessen enligt standardiserat vårdförlopp för makroskopisk hematuri: Från första brevet till en utförd cystoskopi.

Kohorten är uppdelad i två grupper UBC-grupp (verifierad histopatologiskt, n=10) och kontrollgrupp (ingen UBC efter röntgen och cystoskopi, n=10). För att sätta resultaten i sin kontext kommer allmänna medicinska och demografiska frågor ställas i början av intervjun. Deltagarna rekryterades enbart via urologmottagniongen i Uddevalla sjukhus.

Andra studien: Patienters upplevelser av TURB under spinal anestesi?

Kohorten består av informanter med misstänkt UBC och genomgår TURB under spinal anestesi (n=15). För att sätta resultaten i sin kontext kommer allmänna medicinska och demografiska frågor ställas i början av intervjun. Deltagarna rekryterades enbart via urologmottagniongen i Uddevalla sjukhus.

Dataanalys

Det transkriberade materialet från intervjuer kommer att skrivas ut och läsas igenom ett flertal gånger för att få en ökad förståelse för innehållet. Därefter börjar analysprocessen med att

Etikprövningsmyndigheten
2022-01055-01-229278
2022-02-23

identifiera relevanta meningsbärande enheter. Därefter kommer dessa enheter att kodas utifrån innehåll. Koderna skall analyseras för att bilda underkategorier där likheter och skillnader identifieras. Från det latenta kan innehållet av ett övergripande tema urskiljas. För att belysa budskapet i studiens resultat kommer relevanta meningar ur intervjuer citeras.

Etiska överväganden

Studien skall genomföras i enlighet med principerna för forskning som involverar människor som uttrycks i Helsingforsdeklarationen och riktlinjerna för god klinisk praxis [14]. Alla informanter skall informeras muntligt och skriftligt om studien, och vad ett deltagande innebär. Informanterna kommer ha möjlighet att ställa frågor. Informanter skall få information om att deltagandet är frivilligt och om att de när som helst under intervjun kunde välja att avbryta sin medverkan utan att ange orsak.

De konsekutiva 30/15 informanter som ger skriftligt informerat samtycke till att delta kommer att intervjuas. Samtliga ljudupptagningar skall behandlas konfidentiellt och intervjuerna skall kodas för att avidentifiera informanterna. All information skall även samlas in konfidentiellt enligt Dataskyddsförordningen, GDPR, (EU2016/679).

Etikprövningsmyndigheten
2022-01055-01-229278
2022-02-23

Referenser

1. Witjes, J.A., et al., *European Association of Urology Guidelines on Muscle-invasive and Metastatic Bladder Cancer: Summary of the 2020 Guidelines.* European Urology, 2021. **79**(1): p. 82-104.
2. Burger, M., et al., *Epidemiology and risk factors of urothelial bladder cancer.* Eur Urol, 2013. **63**(2): p. 234-41.
3. Shapley, M., et al., *Positive predictive values of >/=5% in primary care for cancer: systematic review.* Br J Gen Pract, 2010. **60**(578): p. e366-77.
4. Hansen, R.P., et al., *Time intervals from first symptom to treatment of cancer: a cohort study of 2,212 newly diagnosed cancer patients.* BMC Health Serv Res, 2011. **11**: p. 284.
5. Nilbert, M., et al., *Diagnostic pathway efficacy for urinary tract cancer: population-based outcome of standardized evaluation for macroscopic haematuria.* Scand J Urol, 2018. **52**(4): p. 237-243.
6. Chung, J., et al., *Assessment of quality of life, information, and supportive care needs in patients with muscle and non-muscle invasive bladder cancer across the illness trajectory.* Support Care Cancer, 2019. **27**(10): p. 3877-3885.
7. Riba, M.B., et al., *Distress Management, Version 3.2019, NCCN Clinical Practice Guidelines in Oncology.* J Natl Compr Canc Netw, 2019. **17**(10): p. 1229-1249.
8. Zimmermann, K., et al., *Health-related quality of life in bladder cancer patients: general and cancer-specific instruments. Part 1.* Curr Opin Urol, 2021. **31**(4): p. 297-303.
9. Jung, A., et al., *Health-related quality of life among non-muscle-invasive bladder cancer survivors: a population-based study.* BJU Int, 2020. **125**(1): p. 38-48.

Etikprövningsmyndigheten
2022-01055-01-229278
2022-02-23

10. Smith, A.B., et al., *Impact of bladder cancer on health-related quality of life.* BJU Int, 2018. **121**(4): p. 549-557.

11. Basch, E., *Patient-Reported Outcomes - Harnessing Patients' Voices to Improve Clinical Care.* N Engl J Med, 2017. **376**(2): p. 105-108.

12. Graneheim, U.H., B.M. Lindgren, and B. Lundman, *Methodological challenges in qualitative content analysis: A discussion paper.* Nurse Educ Today, 2017. **56**: p. 29-34.

13. Gillham, B., *Forskningsintervjun: tekniker och genomförande.* . 2008: Studentlitteratur.

14. *World Medical Association Declaration of Helsinki: ethical principles for medical research involving human subjects.* Jama, 2013. **310**(20): p. 2191-4.

Etikprövningsmyndigheten
2022-01055-01-229278
2022-02-23

Patienters upplevelse av diagnosprocessen enligt standardiserat vårdförlopp för makroskopisk hematuri

En kvalitativ intervjustudie

Projektplan

Etikprövningsmyndigheten
2022-01055-01-262996
2022-05-05

Bakgrund

Urinblåsecancer (UBC) är den 7:e vanligaste cancerformen hos män i världen, medan den är den 11:e när båda könen beaktas [1]. De största riskfaktorerna för BC är rökning, och hög ålder [2]. Makroskopisk hematuri (blod i urinen) är det vanligaste symtomet för UBC [3]. Generellt sett kan en cancerdiagnosfördröjning ha en negativ inverkan på prognosen [4]. Därför implementerades i Sverige under 2016 ett standardiserat vårdförlopp (SVF) som ett snabbspår för patienter med misstänkt UBC, främst på grund av makroskopisk hematuri [5]. Ambitionen var att patienter (50 år eller äldre) ska uppleva en välorganiserad och professionell vård, oavsett var i landet patienter söker vård, samt för att minska ledtider [6]. SVF består av en enhetlig utredning inklusive cystoskopi och skiktröntgen (computed tomography-urography) [6]. För att minska tiden från första symtom till behandling var avsedd ledtid för utredning från remiss till diagnostiska åtgärden (resektion av urinblåsan TUR) 13 dagar.

Etikprövningsmyndigheten
2022-01055-01-262996
2022-05-05

Handläggning av makroskopisk hematuri med att ha i åtanke att man kan ha UBC är naturligtvis en stressande process [7]. Att patienter får vänta länge för att komma till specialiserad vård är ett känt fenomen inom den svenska sjukvården, vilket gör det ännu viktigare att undersöka påverkan av så snabb handläggning på patienternas mående och ifall de upplever situationen som onödigt stressigt.

Även om livskvaliteten (QoL) för UBC-patienter har beskrivits väl i litteraturen, saknas fortfarande en fullständig beskrivning av patienternas upplevelser under diagnosprocessen [8-10]. Att utföra kvalitetsbedömningar i klinisk praxis har visat sig inte bara öka vårdgivarens och patientens medvetenhet om behandlingsrelaterade frågor, utan kan förbättra kommunikationen mellan patienter och vårdpersonal, samt förbättra patienternas överlevnad [11]. Såvitt vi vet har

inga intervjubaserade studier utförts för individer med makroskopisk hematuri som genomgår en snabb diagnosprocess som SVF.

Syfte

Denna intervjustudie syftar till att beskriva hur patienter med makroskopiskt hematuri upplever det standardiserade vårdförloppet för diagnosticering av UBC samt beskriva hur patienter upplever det diagnostiska ingreppet TUR.

Metod

En intervjustudie planeras för att utforska patienters upplevelse av SVF för makroskopisk hematuri. I resten av detta dokument kommer patienter att kallas informanter. Data kommer att analyseras genom innehållsanalys med induktiv ansats enligt Lundman & Graneheim [12]. Denna metod är en relevant metod i kvalitativa studier där både det manifesta och latenta budskapet kan belysas och fokus ligger på att beskriva variationer i texten och identifiera mönster [12]. Enligt Gillham är en intervju en vedertagen metod för att få en direkt insyn av informanters upplevelser, känslor och tankar av det studerade fenomenet (den snabba processen av diagnostisering av eventuell cancer efter att patienter ser blod i urinen) [13].

Denna studie kommer att baseras på individuella intervjuer med en inledande öppen fråga: *Kan du börja berätta från att du första gången fick blod i urinen*? Ett krav för en lyckad induktiv ansats är att materialet skall bearbetas förutsättningslöst så att informanternas upplevelser hamnar i fokus. Intervjuer ska genomföras fysiskt, via telefon eller videosamtal och varar i cirka 35–45. Intervjuer kommer att spelas in och transkriberas därefter ordagrant.

Etikprövningsmyndigheten
2022-01055-01-262996
2022-05-05

Inklusionskriterier

Deltagarna ska vara 50 år eller äldre och ha genomgått SVF för makroskopisk hematuri.

Datainsamling

Kohorten bestå av 15 patienter. För att sätta resultaten i sin kontext kommer allmänna medicinska och demografiska frågor ställas i början av intervjun. Deltagarna skall rekryteras enbart via urologmottagniongen i Uddevalla sjukhus.

Data-analys

Det transkriberade materialet från intervjuer kommer att skrivas ut och läsas igenom ett flertal gånger för att få en ökad förståelse för innehållet. Därefter börjar analysprocessen med att identifiera relevanta meningsbärande enheter. Därefter kommer dessa enheter att kodas utifrån innehåll. Koderna skall analyseras för att bilda underkategorier där likheter och skillnader identifieras. Från det latenta kan innehållet av ett övergripande tema urskiljas. För att belysa budskapet i studiens resultat kommer relevanta meningar ur intervjuer citeras.

Etikprövningsmyndigheten
2022-01055-01-262996
2022-05-05

Etiska överväganden

Studien skall genomföras i enlighet med principerna för forskning som involverar människor som uttrycks i Helsingforsdeklarationen och riktlinjerna för god klinisk praxis [14]. Alla informanter skall informeras muntligt och skriftligt om studien, och vad ett deltagande innebär. Informanterna kommer ha möjlighet att ställa frågor. Informanter skall få information om att deltagandet är frivilligt och om att de när som helst under intervjun kan välja att avbryta sin medverkan utan att ange orsak.

De konsekutiva informanter som ger skriftligt informerat samtycke till att delta kommer att intervjuas. Samtliga ljudupptagningar skall behandlas konfidentiellt och intervjuerna skall kodas för att avidentifiera informanterna. All information skall även samlas in konfidentiellt enligt Dataskyddsförordningen, GDPR, (EU2016/679).

Etikprövningsmyndigheten
2022-01055-01-262996
2022-05-05

Referenser

1. Witjes, J.A., et al., *European Association of Urology Guidelines on Muscle-invasive and Metastatic Bladder Cancer: Summary of the 2020 Guidelines.* European Urology, 2021. **79**(1): p. 82-104.
2. Burger, M., et al., *Epidemiology and risk factors of urothelial bladder cancer.* Eur Urol, 2013. **63**(2): p. 234-41.
3. Shapley, M., et al., *Positive predictive values of >/=5% in primary care for cancer: systematic review.* Br J Gen Pract, 2010. **60**(578): p. e366-77.
4. Hansen, R.P., et al., *Time intervals from first symptom to treatment of cancer: a cohort study of 2,212 newly diagnosed cancer patients.* BMC Health Serv Res, 2011. **11**: p. 284.
5. Abuhasanein, S., et al., *Standardized Care Pathways for Patients with Suspected Urinary Bladder Cancer: The Swedish Experience.* Scandinavian Journal of Urology, 2022.
6. Nilbert, M., et al., *Diagnostic pathway efficacy for urinary tract cancer: population-based outcome of standardized evaluation for macroscopic haematuria.* Scand J Urol, 2018. **52**(4): p. 237-243.
7. Chung, J., et al., *Assessment of quality of life, information, and supportive care needs in patients with muscle and non-muscle invasive bladder cancer across the illness trajectory.* Support Care Cancer, 2019. **27**(10): p. 3877-3885.
8. Zimmermann, K., et al., *Health-related quality of life in bladder cancer patients: general and cancer-specific instruments. Part 1.* Curr Opin Urol, 2021. **31**(4): p. 297-303.
9. Jung, A., et al., *Health-related quality of life among non-muscle-invasive bladder cancer survivors: a population-based study.* BJU Int, 2020. **125**(1): p. 38-48.
10. Smith, A.B., et al., *Impact of bladder cancer on health-related quality of life.* BJU Int, 2018. **121**(4): p. 549-557.
11. Basch, E., *Patient-Reported Outcomes - Harnessing Patients' Voices to Improve Clinical Care.* N Engl J Med, 2017. **376**(2): p. 105-108.
12. Graneheim, U.H., B.M. Lindgren, and B. Lundman, *Methodological challenges in qualitative content analysis: A discussion paper.* Nurse Educ Today, 2017. **56**: p. 29-34.
13. Gillham, B., *Forskningsintervjun: tekniker och genomförande.* . 2008: Studentlitteratur.
14. *World Medical Association Declaration of Helsinki: ethical principles for medical research involving human subjects.* Jama, 2013. **310**(20): p. 2191-4.

Etikprövningsmyndigheten
2022-01055-01-262996
2022-05-05

VÄSTRA GÖTALANDSREGIONEN
NU-SJUKVÅRDEN

Samtycke till att delta i studien

ID

Jag har fått muntliga och skriftliga informationen om vad ett deltagande innebär och har haft möjlighet att ställa frågor. Jag får behålla den skriftliga informationen.

☐ Jag samtycker till att delta i studien *” Patienters upplevelse av diagnosprocessen enligt standardiserat vårdförlopp för makroskopisk hematuri (blod i urinen). En kvalitativ intervjustudie”* och samtycker till att den information jag lämnar behandlas konfidentiellt och enbart används för studiens syfte. Jag samtycker även till att den intervju som genomförs spelas in.

Etikprövningsmyndigheten
2022-01055-01-229279
2022-02-23

Namnförtydligande

____

Plats och datum

____

Underskrift

____

Ansvarig i NU-sjukvården: Suleiman Abuhasanein, Specialistläkare
Telefon: 010-435 3572
E-post: suleiman.abuhasanein@vgregion.se

Studie: Patienters upplevelse av diagnosprocessen enligt standardiserat vårdförlopp för makroskopisk hematuri (blod i urinen) En intervjustudie

Information till forskningspersonerna

Vi vill fråga dig om du vill delta i ett forskningsprojekt. I det här dokumentet får du information om projektet och om vad det innebär att delta.

Vad är det för projekt och varför vill ni att jag ska delta?

Urinblåsecancer är en av de vanligaste cancerform, och blod i urinen (makroskopisk hematuri) är det vanligaste symtomet för den. Generellt sett kan en fördröjning av diagnosen vid cancer ha en negativ inverkan. Därför har man utfört ett standardiserat vårdförlopp (SVF) som är ett snabbspår för patienter med blod i urinen, med ambitionen att förbättra och försnabba handläggning.

Denna handläggning -med att ha i åtanke att man kan ha cancer- är naturligtvis en stressande process. Även om livskvaliteten för urinblåscancerpatienter har beskrivits väl, saknas fortfarande en fullständig beskrivning av patienternas upplevelser under diagnosprocessen.

Vem är forskningshuvudman?

Med forskningshuvudman menas den organisation som är ansvarig för studien. Forskningshuvudman för projektet är Västra Götalandsregionen.

Etikprövningsmyndigheten
2022-01055-01-266135
2022-05-05

Hur går studien till?

Om du väljer att delta i studien kommer du att bli intervjuad 30–60 minuter. Intervjuer ska genomföras antingen per telefon eller fysisk. Intervjuer kommer att spelas in på ljud och transkriberades därefter ordagrant. Intervjun inleds med en öppen fråga: Vill du berätta om när du började se blod i urinen? Sedan får du berätta fritt om din upplevelse av vård hantering av ditt ärende efter det.

Det kommer inte att innebära några ytterligare besök, utöver den vanliga vården. Vi kommer efter ditt besök att läsa i din journal för att inhämta relevanta information.

Möjliga följder och risker med att delta i studien

Ditt deltagande kommer inte att innebära några fysiska risker. Vi kommer att behandla dina data konfidentiellt och så att ingen obehörig ska kunna ta del av dem.

Vad händer med mina uppgifter?

Projektet kommer att samla in och registrera information om dig. De svar du anger i intervjun och journalläsningen kommer att sparas i en databas i kodad form. Kodnyckeln kommer endast att vara tillgänglig för studieansvarig. Dessa kommer endast att ha tillgång till kodade data, och kommer alltså inte att kunna koppla dem till just dig.

Dina svar och dina resultat kommer att behandlas så att inga obehöriga kan ta del av dem. Ansvarig för dina personuppgifter är Västra Götalandsregionen. Enligt EU:s dataskydds-förordning har du rätt att kostnadsfritt få ta del av de uppgifter om dig som hanteras i studien, och vid behov få eventuella fel rättade. Du kan också begära att uppgifter om dig raderas samt att behandlingen av dina personuppgifter begränsas. Om du vill ta del av uppgifterna ska du kontakta studieansvarig (se nedan!).

Dataskyddsombud nås på sahlgrenska.univeristetsjukhuset.dso@vgregion.se. Om du är missnöjd med hur dina personuppgifter behandlas har du rätt att ge in klagomål till Datainspektionen, som är tillsynsmyndighet.

Försäkring och ersättning
Du är försäkrad genom Patientförsäkringen. Någon ersättning för deltagande i studien utgår inte.

Deltagandet är frivilligt
Ditt deltagande är frivilligt och du kan när som helst välja att avbryta deltagandet. Om du väljer att inte delta eller vill avbryta ditt deltagande behöver du inte uppge varför och det kommer inte heller att påverka din framtida vård eller behandling. Om du vill avbryta ditt deltagande ska du kontakta den ansvariga för studien (se nedan!).

Ansvariga för studien

Viola Lindberg Nyman, PhD, Forskningsledare/ FoU- NU/sjukvården. Lecturer at Institute of Health and Care Sciences, University of Gothenburg. Adj. Lecturer at Department of Health sciences, University West, Trollhättan. Tfn: 010-4356895
E-post: viola.nyman@vgregion.se

Suleiman Abuhasanein, Specialistläkare, urologsektionen/kirurgkliniken, NU-sjukvården, doktorand i Göteborgs universitet. Tfn: 010–4353572.
E-post: suleiman.abuhasanein@vgregion.se

Etikprövningsmyndigheten
2022-01055-01-266135
2022-05-05

VÄSTRA GÖTALANDSREGIONEN
NU-SJUKVÅRDEN

Studie: Patienters upplevelse av diagnosprocessen enligt standardiserat vårdförlopp för makroskopisk hematuri (blod i urinen) En intervjustudie

Information till forskningspersonerna
Vi vill fråga dig om du vill delta i ett forskningsprojekt. I det här dokumentet får du information om projektet och om vad det innebär att delta.

Vad är det för projekt och varför vill ni att jag ska delta?
Urinblåsecancer är en av de vanligaste cancerform, och blod i urinen (makroskopisk hematuri) är det vanligaste symtomet för den. Generellt sett kan en fördröjning av diagnosen vid cancer ha en negativ inverkan. Därför har man utfört ett standardiserat vårdförlopp (SVF) som är ett snabbspår för patienter med blod i urinen, med ambitionen att förbättra och försnabba handläggning. SVF består av en enhetlig utredning inklusive cystoskopi och skiktröntgen.
Denna handläggning -med att ha i åtanke att man kan ha cancer- är naturligtvis en stressande process. Att patienter får vänta länge för att komma till specialiserad vård är ett känt fenomen inom sjukvården, vilket gör det ännu viktigare att undersöka påverkan av så snabb handläggning på patienternas mående och ifall de upplever situationen som onödigt stressigt. Även om livskvaliteten för urinblåscancerpatienter har beskrivits väl, saknas fortfarande en fullständig beskrivning av patienternas upplevelser under diagnosprocessen.

Etikprövningsmyndigheten
2022-01055-01-292390
2022-07-12

Vem är forskningshuvudman?
Med forskningshuvudman menas den organisation som är ansvarig för studien. Forskningshuvudman för projektet är Västra Götalandsregionen. Dessutom är studien godkänd av Etikprövningsmyndigheten, dnr 2022-01055-01.

Hur går studien till?
Om du väljer att delta i studien kommer du att bli intervjuad 30–60 minuter. Intervjuer ska genomföras antingen per telefon eller fysisk. Intervjuer kommer att spelas in på ljud och transkriberades därefter ordagrant. Intervjun inleds med en öppen fråga: Vill du berätta om när du började se blod i urinen? Sedan får du berätta fritt om din upplevelse av vård hantering av ditt ärende efter det.
Det kommer inte att innebära några ytterligare besök, utöver den vanliga vården. Vi kommer efter ditt besök att läsa i din journal för att inhämta relevanta information.

Möjliga följder och risker med att delta i studien
Ditt deltagande kommer inte att innebära några fysiska risker. Vi kommer att behandla dina data konfidentiellt och så att ingen obehörig ska kunna ta del av dem.

Vad händer med mina uppgifter?
Projektet kommer att samla in och registrera information om dig. De svar du anger i intervjun och journalläsningen kommer att sparas i en databas i kodad form. Kodnyckeln kommer endast att vara tillgänglig för studieansvarig. Dessa kommer endast att ha tillgång till kodade data, och kommer alltså inte att kunna koppla dem till just dig.
Dina svar och dina resultat kommer att behandlas så att inga obehöriga kan ta del av dem. Ansvarig för dina personuppgifter är Västra Götalandsregionen. Enligt EU:s dataskydds-förordning har du rätt att kostnadsfritt få ta del av de uppgifter om dig som hanteras i studien,

Ansvarig för studien: Suleiman Abuhasanein, Specialistläkare
Telefon: 010-435 3572
E-post: suleiman.abuhasanein@vgregion.se

och vid behov få eventuella fel rättade. Du kan också begära att uppgifter om dig raderas samt att behandlingen av dina personuppgifter begränsas. Om du vill ta del av uppgifterna ska du kontakta studieansvarig (se nedan!).
Dataskyddsombud nås på sahlgrenska.univeristetsjukhuset.dso@vgregion.se. Om du är missnöjd med hur dina personuppgifter behandlas har du rätt att ge in klagomål till Integritetsskyddsmyndigheten (www.imy.se), som är tillsynsmyndighet.

Försäkring och ersättning
Du är försäkrad genom Patientförsäkringen. Någon ersättning för deltagande i studien utgår inte.

Deltagandet är frivilligt
Ditt deltagande är frivilligt och du kan när som helst välja att avbryta deltagandet. Om du väljer att inte delta eller vill avbryta ditt deltagande behöver du inte uppge varför och det kommer inte heller att påverka din framtida vård eller behandling. Om du vill avbryta ditt deltagande ska du kontakta den ansvariga för studien (se nedan!).

Etikprövningsmyndigheten
2022-01055-01-292390
2022-07-12

Ansvariga för studien

Viola Lindberg Nyman, PhD, Forskningsledare/ FoU- NU/sjukvården. Lecturer at Institute of Health and Care Sciences, University of Gothenburg. Adj. Lecturer at Department of Health sciences, University West, Trollhättan. Tfn: 010-4356895
E-post: viola.nyman@vgregion.se

Suleiman Abuhasanein, Specialistläkare, urologsektionen/kirurgkliniken, NU-sjukvården, doktorand i Göteborgs universitet. Tfn: 010–4353572.
E-post: suleiman.abuhasanein@vgregion.se

Etikprövningsmyndigheten
2022-01055-01-292389
2022-07-12

Intervjuguide

Jag har nu startat inspelningen av intervju med XX, godkänner du att samtalet spelas in? Du kan när som helst under inspelningen välja att avbryta ditt deltagande. De uppgifter du delger oss kommer att behandlas konfidentiellt, innebärande att obehöriga inte tillåts se/höra materialet, godkänner du detta?

Bakgrundsfrågor:
Är det något du undrar över innan vi börjar intervjun?
Är du frisk annars?
Tar du några mediciner? Vilka?
Har du någon cancersjukdom? Vilken?
Har du någon sjukdom i urinvägarna? I njurar? I prostata? Vilka?

Semistrukturerade frågor:
Börja berätta från när du såg blod i urinen
Kan du beskriva hur du kände när du såg blod i urinen? Vad tänkte du på just då?
Varför? Utveckla det mera?
Vad har du för generell uppfattning om utredning för blod i urinen?
Vilka erfarenheter har du av urinblåsecancer?

Hur kom du till kontakt med vården? VC eller direkt till oss?
Upplever du att du kom till undersökning inom lagom tid?
Ser du några fördelar med att komma så här snabbt till undersökningen?
Ser du några nackdelar med att komma så här snabbt till undersökningen?
Lämpligt när i så fall?
Hur fick du diagnosen urinblåsecancer? Vad det förväntat eller kom det plötsligt? Utveckla?
Upplever du info du fick tillräckliga?
Beskriv hur var det när du genomgick cystoskopi och operationen där man hyvlade tumören?
Tror du att du fick den vård som du behöver?
Är det något annat du vill tillägga innan vi avslutar samtalet?

Nu avslutar jag inspelningen av intervju med dig, tack så mycket för din medverkan!

Ansvarig för studien: Suleiman Abuhasanein, Specialistläkare
Telefon: 010-435 3572
E-post: suleiman.abuhasanein@vgregion.se

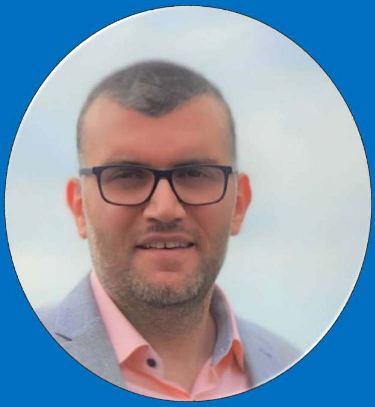

Suleiman Abuhasanein

Urolog

Specialistläkare urologi, ansvarig för urinblåsecancer-verksamhet i urolog-sektionen NU-sjukvården, och doktorand vid Institute of Clinical Sciences / Sahlgrenska Academy.

Etikprövningsmyndigheten
2022-01055-01-229280
2022-02-23

KONTAKTINFO

Telefon
+46 729 000 305

Epost
Suleiman.abuhasanein@gmail.com

Adress
Einars väg 3, 451 98, Uddevalla

Kompetenser

Mycket arbetsvillig
Statistik och siffror
Lär mig nya uppgifter snabbt
Utåtriktad och social

UTBILDNING

Damaskus universitet, Syrien, 2001–2007
Läkarprogrammet

Göteborgs universitet, Sverige, 2020-
Doktorand vid Institute of Clinical Sciences / Sahlgrenska Academy

ARBETSERFARENHET

Urologsektionen, NU-sjukvården, 2021-
Specialistläkare-urologi

NU-sjukvården, 2018–2021
ST-läkare, urologi

NU-sjukvården, 2017–2018
AT-läkare

Ljungskile VC 2016–2017
Underläkare

NU-sjukvården, 2015–2016
Underläkare, urologi

Damaskus sjukhus 2008–2013
ST-läkare, urologi

Damaskus sjukhus 2007–2008
Underläkare

Vetenskapliga arbeten

1- Abuhasanein S, et al. A rare case of a necrotized urethral prolapse in a postmenopausal woman with acute urinary retention. Low Urin Tract Symptoms. 2021 Oct;13(4):505-508. doi: 10.1111/luts.12381. Epub 2021 May 5. PMID: 33951749.

Konferenser & finansiering

- Urologidagarna-2019. Deltagit med tre vetenskapliga papper (SVF-tider för urinblåsecancer (UBC), UBC-T1, och röntgen och UBC).
- SIU43 i Dubai 2021 deltagit med ett abstract reresektion vid T1-UBC)
- Fick stipendium av Anders och Carl Erikssons Fond för Medicinsk forskning 2021 för min forskning inom urinblåsecancer

Curriculum Vitae

Viola Nyman

Home address: Kungsgatan 1A, 462 33 Vänersborg, Sweden.
Phone: +46705 221 973
Birth date: 17-12-1961, Sodankyla, Finland.
Employment: Midwife at Förlossningsavdelning 36 NÄL and Research director at Department of Research and Development NU-Hospital Group
Lärketorpsvägen, SE-461 85 Trollhättan Sweden
PhD, Adj. Lecturer at Institute of Health and Care Sciences, Sahlgrenska Academy, University of Gothenburg, Sweden
Adj. Lecturer at Institute at Health Sciences, University West, Trollhättan, Sweden
Phone +46 10 43 56895 Mob +46 705221973
Mail viola.nyman@vgregion.se

Etikprövningsmyndigheten
2022-01055-01-252224
2022-03-28

Arbetslivserfarenhet

2021	Universitetslektor vid Institutionen för Vårdvetenskap och Hälsa, Sahlgrenska Akademin, Göteborgs Universitet.
2019	Kompetensstöd i utredningen ”Senior Arbetskraft i Västra Götalandsregionen” Diarienummer RS 2018-06570. Utredningens uppdrag från Personalutskottet: Kartlägga, tydliggöra och stärka arbetet kring att locka äldre, erfarna medarbetare att arbeta kvar inom sjukvården.
2016-	Forskningsledare FOU enheten, NU-Sjukvården, Norra Älvsborgs Länssjukhus (NÄL) Trollhättan.
2016-	Adjungerad lektor vid Institutionen för Vårdvetenskap och Hälsa, Sahlgrenska Akademin, Göteborgs Universitet.
2018-	Adjungerad lektor vid Institutionen för Hälsovetenskap, Högskolan Väst, Trollhättan
1997-<br>1999	Barnmorska, Klinisk studie, multicenter RCT, Akzo Nobel.
1995-	Barnmorska – Förlossningsavdelning, BB avd, Gyn avd. Gyn mott. NÄL. tillsvidare
1994	Sjuksköterska, BB och Gyn avd. NÄL.
1993	Sjuksköterska, Barn avd, Medicin avd. NÄL.
1992-<br>1993	Utlandsvistelse, Vancouver Kanada.
1987-	Operationssjuksköterska. Lasarettet i Vänersborg (LIV). ÖNH mott, Barnklinik, LIV/NÄL, Kvinnokliniken, NÄL.
1987	Sjuksköterska, Torpa Vårdcentral, Vänersborg.

Utbildning

2015	Avhandling, Institutionen för Vårdvetenskap och Hälsa,<br>Titel: From the first encounter to management of childbirth - An insider action research in a labour ward world. Sahlgrenska Akademin, GU.<br>Huvudhandledare: Professor Marie Berg, Bihandledare: Professor Soo Downe, Storbritannien och Professor Terese Bondas, Norge. Filosofie doktorsexamen i Vårdvetenskap och Hälsa, Göteborgs universitet

2008	Magisterexamen i huvudämnet omvårdnad, 30hp, Högskolan Väst
2007	Kandidatexamen i Folkhälsa och hälsopedagogik, 20P, Högskolan Väst
2002	Omvårdnad, Folkhälsa o hälsopedagogik 20P, Högskolan Väst
1995	Barnmorskeexamen, 50P, Vårdskolan i Göteborg
1991	Sjuksköterskeexamen, kirurgi och medicin, 40P, Vårdhögskolan, Vänersborg
1987	Sjuksköterskeexamen, inriktning operation, 80P, Vårdhögskolan, Vänersborg

Övrigt

2016	Så skriver du en kommunikationsplan, en dags kurs, GU
2016	Projektledning inom vård och omsorg, 8-9 juni, Stockholm
2005-2008	Intervju och samtalsmetodik, personlighetsutveckling, psykopatologi, NU-sjukvården ledare: Psykolog Anki Goldberg, Org. Förlossningsrädsla
2009	Case metodik, 2 dagar, NU-sjukvården
2009	The Art and Science of Midwifery 7,5 hp, Sahlgrenska Akademin GU.
1999	Klinisk läkemedelsprövning och GCP, 2 dagar, Västra Götaland regionen
1998	Engelska 20P, Högskolan i Trollhättan/Uddevalla.
1993	Engelska, Vancouver, Kanada.

Språk

Svenska
Finska
Engelska

Etikprövningsmyndigheten
2022-01055-01-252224
2022-03-28

Referenser

Ingela Lundgren, Professor i Vårdvetenskap och Hälsa, Reproduktiv och perinatal hälsa, Sahlgrenska Akademi, Göteborgs Universitet, Göteborg, Sverige
Tel: +4631 78 660 52
Ingela.lundgren@fhs.gu.se

Helen Elden, Docent i Vårdvetenskap och Hälsa, Reproduktiv och perinatal hälsa, Sahlgrenska Akademi, Göteborgs Universitet, Göteborg, Sverige
Tel: +4631 70 288 78 82
Helen.elden@gu.se

Annika Månebacke Wändel, Avdelningschef Förlossningsavdelning NÄL, Trollhättan, Sverige
Tel: +4610 43 503 64
annika.wandel@vgregion.se

Meritportfölj

Portfölj 1: Vetenskapliga meriter
Publikationer

7. How do midwives facilitate women to give birth during physiological second stage of labour? A systematic review. Healy M, Nyman V, Spence D, Otten RHJ, Verhoeven CJ. PLoS One. 2020 Jul 28;15(7):e0226502. doi: 10.1371/journal.pone.0226502. eCollection 2020. PMID: 32722680

6. Verhoeven C, Spence D, Nyman V, Otten, R, Healy M. How do midwives facilitate women to give birth during physiological second stage of labour? A protocol for a systematic review. SYSR-D-18-00343R2

5. Nyman V, Roshani L, Berg M, Bondas T, Downe S, Dencker A. *Routine interventions in childbirth before and after initiation of action research*. Sexual Reproductive Healthcare. 2017; 11:86–90.

4. Nyman V, Berg M, Downe S, Bondas T. *Insider Action research as an approach and a method – exploring encounters from within a birthing context*. Action Research. 2016; 14(2):217-233.

3. Nyman V, Bondas T, Downe S, Berg M. *Glancing beyond or being confined to routines: Labour ward midwives' responses to change as a result of action research*. Midwifery. 2013 Jun; 29(6):573-8.

2. Nyman V, Downe S, Berg M. *Waiting for permission to enter the labour ward world: First time parents' experiences of the first encounter on a labour ward*. Sexual & Reproductive Healthcare. 2011; 2(3):129-34.

1. Nyman V, Prebensen Å, Flensner G. (2010). *Obese women's experiences of encounters with midwives and physicians during pregnancy and childbirth*. Midwifery, 26(4), 424-429.

Forskarkurser

2012	Action Research in Nursing Science, 8,0 hp, Kuopio, University of Eastern Finland
2011	Introduction to research, 15 hp, Göteborgs Universitet, Sverige.
2011	Interpretive Description, 2 hp, Göteborgs Universitet, Sverige
2010	Action Research, 7,5 hp Högskolan Borås, Sverige

Etikprövningsmyndigheten
2022-01055-01-252224
2022-03-28

Vetenskapligt CV
Presentationer vid vetenskapliga konferenser

2019 Muntlig presentation (abstraktbok) *Midwives' practice during the second stage of physiological labour: A systematic review.* The 21st Congress of the Nordic Federation of Midwives, held in Reykjavik, Iceland, 2-4 May 2019

2017 Muntlig presentation (abstraktbok). *From the first encounter to management of childbirth – An insider action research in a labour ward world*. Midwives making a difference in the world. The 31st ICM Triennial Congress, 18-22 June, Toronto, Canada.

2017 Poster presentation (abstraktbok). *I know I'm fat, you don't have to tell me*. Midwives making a difference in the world. The 31st ICM Triennial Congress, 18-22 June, Toronto, Canada.

2014 Muntlig presentation. *Midwives´ experiences of action research and the nature of the first encounter on a hospital based labour ward in Sweden*. Optimizing childbirth across Europe - an interdisciplinary maternity care conference. 9-10 April 2014, Brussels, Belgium. Part of COST Action IS0907: Childbirth Cultures, Concerns & Consequences: Creating a dynamic EU framework for Optimal maternity care, Belgium.

Etikprövningsmyndigheten
2022-01055-01-252224
2022-03-28

2014 Poster presentation (abstraktbok). *Do I need to change? Midwives´experiences of action research and the first encounter on labour ward in Sweden*, The Nordic Conference on Advances in Health Care Sciences Research, 12-13 November, University of Turku, Åbo, Finland.

2013 Muntlig presentation (abstraktbok). *The first encounter on a labor ward - Action research for a change*. 19th Nordic Midwifery Congress – Nordic and Global Challenges 13–15 June 2013, Oslo, Norge.

2013 Muntlig presentation (abstraktbok). *The first encounter on a labour ward - Action Research for a change.* 8th Normal Labour and Birth Conference 5th-7th June 2013 at the Grange Hotel Conference Centre, Grange-over-Sands, Cumbria, England.

2012 Muntlig presentation. *Glancing beyond or being confined to routines: labour ward midwives' responses to change as a result of action research*. BFin Research Workshop and Research Project Development Advanced Research in Childbearing, Maternal and Neonatal care and Transcultural perspectives, University of Nordland, Faculty of Professional Studies and University of Tromsø, Institute of Health and Caring Science in collaboration with BFiN: Childbearing in Europe-the qualitative research network in childbearing. GU, Göteborg, Sverige.

2008 Poster presentation (abstraktbok). *Överviktiga kvinnors upplevelse av barnmorskors och läkares bemötande.* SFOG veckan, Skövde, Sverige.

Portfölj 2: Pedagogiska meriter
Högskolepedagogiska utbildningar/kurser

2017	Behörighetsgivande Högskolepedagogik HPE101, GU, 7,5 hp
2016	Handledning i forskarutbildning, HPE 201, GU, 5,0 hp.
2004	Handledarutbildning 5P Högskolan Väst.

Pedagogiskt CV
Utvecklingsarbete och pedagogisk forskning

2021	Universitetslektor Barnmorskeprogrammet, Vårdvetenskap och Hälsa, SA, GU
2016-	Adjungerad
2016	Handledning Kliniskt Basår för sjuksköterskor, Nu-sjukvården
2009- 2012	Klinisk aktionsforskning. Artikel i tidskriften: Action Research.

Pedagogisk meritering - reflektion
Efter många års kliniskt arbete inom vården ville jag försöka utveckla vår kliniska kompetens genom att utveckla arbetsrutiner i syfte att patienten ska få bästa tänkbara vård och upplevelse av behandling och bemötande. Mitt avhandlingsarbete var aktionsforskning i egen organisation som handlade om att beskriva och samtidigt utveckla bemötande och förhållningssätt på en förlossningsavdelning. Att göra aktionsforskning var ett sätt att försöka länka praktisk och akademisk kunskap, vilket krävs för att öka användandet av evidensbaserad kunskap i kliniskt arbete. Detta fyraåriga kollegiala processarbete var en pedagogisk utmaning och lärande. Att vara aktionsforskare innebar kontinuerliga föreläsningar och planering av utbildnings- och utvecklingstillfällen för kliniska barnmorskor och läkare. Dessa utvecklingstillfällen handlade om ämnet som innebar att aktivt jobba för att normalisera födandet men också om själva förändringsprocessen. Det medförde att jag fick feedback på vad och hur jag klarade den pedagogiska uppgiften kontinuerligt av kollegor.

Etikprövningsmyndigheten
2022-01055-01-252224
2022-03-28

Undervisning och kursansvar på Barnmorskeprogrammet, SA, GU i kursen Normal graviditet, förlossning och tidigt föräldraskap, samt föreläsningar på Kvinnokliniker i Sverige om hur vi kan jobba med förhållningssätt och rutiner inom förlossningsvården.

Portfölj 3: Ledarskap/akademiskt ledarskap
Övriga ledarskapserfarenheter

2019	SKL Sthlm föreläsning Trygg hela vägen
2018	Referensgrupp för Universitetskanslersämbetet (UKÄ) gällande Sophia hemmet ansökan om utfärdande av Mag examen i Reproduktiv och perinatal hälsa (RPH)
2018	Handledning vetenskapligt arbete för ST arbete, läkare KK NU-sjukvården
2018	Chair för muntlig session under 31e Internationella barnmorskekonferens (ICM Triennial Congress, Toronto, Kananda).

2017-	Handledare för magisterstudenter på GU och doktorand inom Reproduktiv och Perinatal Hälsa, GU. Handledare för magisterstudenter HV, samt bihandledare för doktorander: Anna-Carin Robertz SA, GU; Ragnhild Eikemo, KI, Maria Detlin, HV, Trollhättan.
2016	Utformande av en kontinuerlig veckovis återkommande Vårdvetenskapligt forum (VVF) för sjuksköterskor i NU-sjukvården
2010-2013	Processledare, återges i doktorsavhandling.

Etikprövningsmyndigheten
2022-01055-01-252224
2022-03-28

Signering av etikprövningsansökan

Grundansökan

Forskningshuvudman: Västra Götalandsregionen
Projekttitel: Patienters upplevelse av diagnosprocessen enligt standardiserat vårdförlopp för makroskopisk hematuri En kvalitativ intervjustudie

I och med att ansökan undertecknas intygar du som är behörig företrädare följande:

- Att den information som lämnas i ansökan om etikprövning och samtliga medföljande bilagor är riktig och fullständig.
- Att verksamhetsansvariga i samtliga medverkande verksamheter är informerade om forskningsprojektets innehåll och utförande och att de har samtyckt till att delta i studien.
- Att du säkerställt att det i samtliga medverkande verksamheter finns resurser som garanterar forskningspersonernas säkerhet och integritet vid genomförandet av den forskning som beskrivs i ansökan.
- Att ansvarig forskare ges rätt att företräda huvudmannen i alla framtida kontakter med Etikprövningsmyndigheten som rör detta forskningsprojekt samt ansöka om ändringar i forskningsprojektet.
- Att du tagit del av Etikprövningsmyndighetens information om hantering av personuppgifter på myndighetens webbplats.

Behörig företrädare har signerat.

Signerat av MARIA VIKSTEN ERICSSON (197312164820) 2022-02-21 16:03:38

Etikprövningsmyndigheten
2022-01055-01-239627
2022-02-23

Signering av etikprövningsansökan

Grundansökan

Forskningshuvudman: Västra Götalandsregionen
Projekttitel: Patienters upplevelse av diagnosprocessen enligt standardiserat vårdförlopp för makroskopisk hematuri En kvalitativ intervjustudie

I och med att ansökan undertecknas intygar du som är ansvarig forskare följande:

- Att den information som lämnas i ansökan om etikprövning och samtliga medföljande bilagor är riktig och fullständig.
- Att verksamhetsansvariga i samtliga medverkande verksamheter är informerade om forskningsprojektets innehåll och utförande och att de har samtyckt till att delta i studien.
- Att du säkerställt att det i samtliga medverkande verksamheter finns resurser som garanterar forskningspersonernas säkerhet och integritet vid genomförandet av den forskning som beskrivs i ansökan.
- Att du tagit del av Etikprövningsmyndighetens information om hantering av personuppgifter på myndighetens webbplats.

Ansvarig forskare har signerat.

Signerat av Suleiman Abuhasanein (198312145850) 2022-02-21 14:30:56

Etikprövningsmyndigheten
2022-01055-01-239481
2022-02-23

Signering av etikprövningsansökan administrativt tillägg

Grundansökan

Forskningshuvudman: Västra Götalandsregionen
Projekttitel: Patienters upplevelse av diagnosprocessen enligt standardiserat vårdförlopp för makroskopisk hematuri En kvalitativ intervjustudie

I och med att ansökan undertecknas intygar du som är ansvarig forskare samt du följande:

- Att den information som lämnas i ansökan om etikprövning och samtliga medföljande bilagor är riktig och fullständig.
- Att verksamhetsansvariga i samtliga medverkande verksamheter är informerade om forskningsprojektets innehåll och utförande och att de har samtyckt till att delta i studien.
- Att du säkerställt att det i samtliga medverkande verksamheter finns resurser som garanterar forskningspersonernas säkerhet och integritet vid genomförandet av den forskning som beskrivs i ansökan.
- Att du tagit del av Etikprövningsmyndighetens information om hantering av personuppgifter på myndighetens webbplats.

Ansvarig forskare har signerat.

Signerat av Suleiman Abuhasanein (198312145850) 2022-03-26 07:37:15

Etikprövningsmyndigheten
2022-01055-01-252228
2022-03-28

Signering av etikprövningsansökan komplettering efter beslut

Grundansökan

Forskningshuvudman: Västra Götalandsregionen
Projekttitel: Patienters upplevelse av diagnosprocessen enligt standardiserat vårdförlopp för makroskopisk hematuri. En kvalitativ intervjustudie.

I och med att ansökan undertecknas intygar du som är ansvarig forskare samt du följande:

- Att den information som lämnas i ansökan om etikprövning och samtliga medföljande bilagor är riktig och fullständig.
- Att verksamhetsansvariga i samtliga medverkande verksamheter är informerade om forskningsprojektets innehåll och utförande och att de har samtyckt till att delta i studien.
- Att du säkerställt att det i samtliga medverkande verksamheter finns resurser som garanterar forskningspersonernas säkerhet och integritet vid genomförandet av den forskning som beskrivs i ansökan.
- Att du tagit del av Etikprövningsmyndighetens information om hantering av personuppgifter på myndighetens webbplats.

BankID

Ansvarig forskare har signerat.

Signerat av Suleiman Abuhasanein (198312145850) 2022-05-05 13:50:22

Etikprövningsmyndigheten
2022-01055-01-266139
2022-05-05

Signering av etikprövningsansökan komplettering efter beslut

Grundansökan

Forskningshuvudman: Västra Götalandsregionen
Projekttitel: Patienters upplevelse av diagnosprocessen enligt standardiserat vårdförlopp för makroskopisk hematuri. En kvalitativ intervjustudie.

I och med att ansökan undertecknas intygar du som är ansvarig forskare samt du följande:

- Att den information som lämnas i ansökan om etikprövning och samtliga medföljande bilagor är riktig och fullständig.
- Att verksamhetsansvariga i samtliga medverkande verksamheter är informerade om forskningsprojektets innehåll och utförande och att de har samtyckt till att delta i studien.
- Att du säkerställt att det i samtliga medverkande verksamheter finns resurser som garanterar forskningspersonernas säkerhet och integritet vid genomförandet av den forskning som beskrivs i ansökan.
- Att du tagit del av Etikprövningsmyndighetens information om hantering av personuppgifter på myndighetens webbplats.

Etikprövningsmyndigheten
2022-01055-01-292392
2022-07-12

Ansvarig forskare har signerat.

Signerat av Suleiman Abuhasanein (198312145850) 2022-07-12 10:21:13

Signering av etikprövningsansökan

Grundansökan

Forskningshuvudman: Västra Götalandsregionen
Projekttitel: Patienters upplevelse av diagnosprocessen enligt standardiserat vårdförlopp för makroskopisk hematuri En kvalitativ intervjustudie
Ansvarig forskare: Suleiman Abuhasanein

Jag intygar att jag som disputerad forskare kommer att utöva aktivt överinseende över den forskning som bedrivs i forskningsprojekt.

Handledare har signerat.

Signerat av Viola Maria Katariina Nyman (196112172942)
2022-02-21 15:36:32

Etikprövningsmyndigheten
2022-01055-01-239558
2022-02-23

Avgiftsavisering

Etikprövningsmyndigheten har tagit emot din ansökan med titel Patienters upplevelse av diagnosprocessen enligt standardiserat vårdförlopp för makroskopisk hematuri En kvalitativ intervjustudie om etikprövning. Ansökan har diarienummer 2022-01055-01 vilket alltid ska anges i framtida kontakter i ärendet.

Avgiften för ansökan, som är 5000 kronor, ska omgående betalas in enligt nedan:

- Inbetalning sker till bankgironummer 406-1107
- Vid inbetalning ska OCR-nummer 2022010550137 anges som referens.
- Inga andra bokstäver eller siffror får anges i raden för referens.

Först när ärendet kompletterats enligt ovan kommer vi att påbörja handläggningen.

Etikprövningsmyndigheten
Telefon: 010 - 475 08 00
Webbplats: www.etikprovning.se

Etikprövningsmyndigheten
2022-01055-01-239626
2022-02-21

Begäran om administrativt tillägg

2022-03-25

Sökande forskningshuvudman
Västra Götalandsregionen

Forskare som genomför projektet
Suleiman Abuhasanein

Projekttitel
Patienters upplevelse av diagnosprocessen enligt standardiserat vårdförlopp för makroskopisk hematuri En kvalitativ intervjustudie

Uppgifter om ansökan
Ansökan inkom till Etikprövningsmyndigheten 2022-02-21.

Etikprövningsmyndigheten begär administrativt tillägg av din ansökan om etikprövning enligt följande:

Etikprövningsmyndigheten
2022-01055-01-252223
2022-03-26

Hej

Etikprövningsmyndigheten har tagit emot din ansökan om etikprövning med dnr 2022-01055-01 och titel "Patienters upplevelse av diagnosprocessen enligt standardiserat vårdförlopp för makroskopisk hematuri

En kvalitativ intervjustudie".

Ansökan har validerats och följande administrativa tillägg behöver göras:

1. Vid punkt 7.4 samt 7.5 måste mer utförligare beskrivningar tillföras. Det räcker inte att skriva "se ovan" och "ej aktuellt".

2. Det saknas CV för disputerad forskare som utövar aktivt överinseende över forskningen. Inkom med detta.

Logga in i Ethix och åtgärda det ovan beskrivna senast 2022-04-01. Ansvarig forskare måste signera när tillägget ska skickas in och när myndigheten mottagit det kommer handläggningen att fortsätta.

E-posta eventuella frågor till registrator@etikprovning.se.

Med vänlig hälsning

Sara Thomasdotter Berggren

Handläggare

Etikprövningsmyndigheten

Telefon: 010-475 08 00

Webbplats: www.etikprovning.se

Begäran sänds till

Ansvarig forskare: Suleiman Abuhasanein

Etikprövningsmyndigheten
2022-01055-01-252223
2022-03-26

BESLUT

2022-04-13

Sökande forskningshuvudman
Västra Götalandsregionen

Forskare som genomför projektet
Suleiman Abuhasanein

Projekttitel
Patienters upplevelse av diagnosprocessen enligt standardiserat vårdförlopp för makroskopisk hematuri
En kvalitativ intervjustudie

Uppgifter om ansökan
Ansökan inkom till Etikprövningsmyndigheten 2022-02-21 och blev valid 2022-03-26.

Etikprövningsmyndigheten beslutar enligt nedan.

Etikprövningsmyndigheten
2022-01055-01-262499
2022-04-25

BESLUT

Etikprövningsmyndigheten begär att sökanden kompletterar ansökan enligt följande:

1. Ansökan och forskningsplan är inte kongruenta, vilket innebär att det är oklart hur många studier ansökan avser, hur många informanter som ska inkluderas, nedre åldersgräns för inklusion, när i förhållande till SVF intervjuerna ska genomföras samt huruvida information ska hämtas genom retrospektiv journalgenomgång. Allt detta behöver förtydligas. Vilken information som ev. ska hämtas via journalgranskning ska även framgå.
2. Bakgrunden och argument för studiens värde behöver förtydligas.
3. Beskrivningen av förfarandet med data (anonymisering? pseudonymisering?) måste förtydligas och vara kongruent mellan ansökans olika delar.
4. Forskningspersonsinformation/er för de olika delstudierna ska bifogas ansökan. Dessa bör utformas enligt stödmall på Etikprövningsmyndighetens hemsida (www.etikprovning.se).
5. Intervjuguide(r) ska bifogas.
6. I forskningsplanen uttrycks att "deltagarna rekryterades" - vilket antyder att data redan kan vara insamlat. Stämmer detta? Myndigheten prövar inte redan påbörjad forskning.

Kompletteringen ska ha kommit in till Etikprövningsmyndigheten senast den 2022-05-18.

Etikprövningsmyndigheten tar sedan upp ärendet till prövning vid ett kommande sammanträde. Om kompletteringen inte kommer in i tid kommer myndigheten att pröva ärendet utifrån det underlag som finns nu.

Kompletteringen görs i Ethix.

Beslut om komplettering innebär att ansökan låses upp för redigering. De begärda kompletteringarna görs direkt i respektive fält, systemet kommer markera upp vilka ändringar som gjorts. Ska kompletterande bilagor laddas upp görs detta under rubriken 'Av myndighetsbeslut begärda komplettering'. Markera ändringar i reviderade bilagor. Besvara kompletteringspunkterna och beskriv i avsett fält för att förtydliga vilka ändringar som gjorts. När kompletteringarna är genomförda ska ansökan signeras och skickas in på nytt.

På Etikprövningsmyndighetens vägnar

Åsa Hanna
Ordförande

Beslutet har fattats av följande personer:

Ordförande
Åsa Hanna (rådman)

Ledamöter med vetenskaplig kompetens
Lars-Gunnar Gunnarsson (neurologi, vetenskaplig sekreterare)
Martin Höglund (hematologi, invärtes medicin, vetenskaplig sekreterare)
Kristina Arnrup (odontologi, föredragande)
Peter Appelros (neurologi, geriatrik, registerstudier, epidemiologi)
Yvonne Freund-Levi (geriatrik, psykiatri)
Arja Harila-Saari (barnonkologi)
Janeth Leksell (vårdvetenskap, diabetes)
Margareta Möller (vårdvetenskap)
Ola Nilsson (pediatrik)

Ledamöter som företräder allmänna intressen
Mats Dahlström
Ziita Eriksson
Agne Furingsten
Isak Rutqvist
Inger Trodell Dahl

Beslutet sänds till

Ansvarig forskare: Suleiman Abuhasanein

Forskningshuvudmannens företrädare: Maria Viksten Ericsson

Etikprövningsmyndigheten
2022-01055-01-262499
2022-04-25

BESLUT

2022-06-08

Sökande forskningshuvudman
Västra Götalandsregionen

Forskare som genomför projektet
Suleiman Abuhasanein

Projekttitel
Patienters upplevelse av diagnosprocessen enligt standardiserat vårdförlopp för makroskopisk hematuri.
En kvalitativ intervjustudie.

Uppgifter om ansökan
Ansökan inkom till Etikprövningsmyndigheten 2022-02-21 och blev valid 2022-03-26. Av myndigheten begärd komplettering enligt beslut 2022-04-13 inkom 2022-05-05.

Etikprövningsmyndigheten
2022-01055-01-282524
2022-06-20

Etikprövningsmyndigheten beslutar enligt nedan.

BESLUT

Etikprövningsmyndigheten begär att sökanden kompletterar ansökan enligt följande:

1. Intervjuguide ska bifogas ansökan.
2. Kommer även SVF-UBC-patienter där utredningen varit negativ, dvs ingen urotelial cancer påvisats, att kunna inkluderas i studien?
3. I forskningspersoninformationen ska syftet med studien beskrivas tydligare (det är tydligare beskrivet i ansökans punkt 3.2)
4. I forskningspersoninformationen ska:
a. tillfogas information om att "Studien är godkänd av Etikprövningsmyndigheten, dnr 2022-01055-01".
b. "Datainspektionen" ändras till "Integritetsskyddsmyndigheten (www.imy.se)".

Kompletteringen ska ha kommit in till Etikprövningsmyndigheten senast den 2022-07-13.

Etikprövningsmyndigheten ger ordföranden i uppdrag att fatta beslut i ärendet när kompletteringen har kommit in. Om kompletteringen inte inkommer i tid kommer Etikprövningsmyndigheten att pröva ärendet utifrån det underlag som finns nu.

Kompletteringen görs i Ethix.

Beslut om komplettering innebär att ansökan låses upp för redigering. De begärda kompletteringarna görs direkt i respektive fält, systemet kommer markera upp vilka ändringar som gjorts. Ska kompletterande bilagor laddas upp görs detta under rubriken 'Av myndighetsbeslut begärda komplettering'. Markera ändringar i reviderade bilagor. Besvara

kompletteringspunkterna och beskriv i avsett fält för att förtydliga vilka ändringar som gjorts. När kompletteringarna är genomförda ska ansökan signeras och skickas in på nytt.

På Etikprövningsmyndighetens vägnar

Åsa Hanna
Ordförande

Beslutet har fattats av följande personer:

Ordförande
Åsa Hanna (rådman)

Ledamöter med vetenskaplig kompetens
Martin Höglund (hematologi, invärtes medicin, vetenskaplig sekreterare, föredragande)
Lars-Gunnar Gunnarsson (neurologi, vetenskaplig sekreterare)
Olof Eriksson (molekylär avbildning, radiologi)
Yvonne Freund-Levi (geriatrik, psykiatri)
Mårten Fryknäs (klinisk farmakologi)
Janeth Leksell (vårdvetenskap, diabetes)
Margareta Möller (vårdvetenskap)

Etikprövningsmyndigheten
2022-01055-01-282524
2022-06-20

Ledamöter som företräder allmänna intressen
Ziita Eriksson
Agne Furingsten
Bengt Sandblad
Inger Trodell Dahl

Beslutet sänds till

Ansvarig forskare: Suleiman Abuhasanein

Forskningshuvudmannens företrädare: Maria Viksten Ericsson

BESLUT

2022-07-26

Sökande forskningshuvudman
Västra Götalandsregionen

Forskare som genomför projektet
Suleiman Abuhasanein

Projekttitel
Patienters upplevelse av diagnosprocessen enligt standardiserat vårdförlopp för makroskopisk hematuri.
En kvalitativ intervjustudie.

Uppgifter om ansökan
Ansökan inkom till Etikprövningsmyndigheten 2022-02-21 och blev valid 2022-03-26. Av myndigheten begärd komplettering enligt beslut inkom 2022-07-12.

Etikprövningsmyndigheten beslutar enligt nedan.

BESLUT

Etikprövningsmyndigheten godkänner den forskning som anges i ansökan.

På Etikprövningsmyndighetens vägnar

Maria Wetterstrand Hagström
Ordförande

Beslutet har fattats av följande personer:

Ordförande
Maria Wetterstrand Hagström (rådman)

Beslutet har fattats efter föredragning av vetenskaplig sekreterare
Martin Höglund

Beslutet sänds till

Ansvarig forskare: Suleiman Abuhasanein

Forskningshuvudmannens företrädare: Maria Viksten Ericsson

Etikprövningsmyndigheten
2022-01055-01-295515
2022-07-26
